# Detecting sleep apnea using non-linear measures of heart rate variability

**DOI:** 10.1186/s12931-026-03621-6

**Published:** 2026-03-19

**Authors:** Topi Niemi, Matias Kanniainen, Marjaana Nurmo, Teemu Pukkila, Soroosh Solhjoo, Esa Räsänen

**Affiliations:** 1 Computational Physics Laboratory, Tampere University, P.O. Box 692, Tampere, FI-33014 Finland; 2 F. Edward Hébert School of Medicine, Bethesda, Maryland USA

**Keywords:** Cardiovascular disease, Heart rate variability, Scale-dependent detrended fluctuation analysis, Sleep Apnea, Wearable sensors

## Abstract

**Background:**

Sleep apnea is highly prevalent yet frequently underdiagnosed due to the cost and complexity of polysomnography. Heart rate variability (HRV) offers a scalable alternative for early screening, but conventional HRV metrics often overlook the multi-scale autonomic disturbances characteristic of apnea.

**Methods:**

We evaluated scale-dependent detrended fluctuation analysis (sDFA), which quantifies how heartbeat-interval correlations evolve across temporal scales, using RR-interval data from Sleep Heart Health Study ($n=5804$). The discriminative performance of sDFA was compared with conventional HRV measures across mild, moderate, and severe apnea, and within cardiovascular disease (CVD) subgroups. Propensity score matching was applied for age and body mass index, and analyses were stratified by sex.

**Results:**

Across apnea severity levels, sDFA consistently outperformed conventional HRV measures in discriminating individuals with sleep apnea. Performance gains were particularly evident in severe apnea and remained robust in participants with CVD, a subgroup in which traditional HRV metrics showed reduced discriminative ability. sDFA revealed scale-specific signatures of autonomic dysfunction that were not captured by conventional time- and frequency-domain HRV measures.

**Conclusion:**

Multi-scale analysis of HRV using sDFA enhances the detection of sleep apnea across severity levels and cardiovascular risk profiles. These findings highlight the limitations of conventional HRV metrics and support sDFA as a promising tool for scalable, HRV-based sleep apnea screening, with potential for integration into wearable and ambulatory monitoring systems.

**Supplementary Information:**

The online version contains supplementary material available at 10.1186/s12931-026-03621-6.

## Background

Obstructive sleep apnea (OSA) is one of the most prevalent sleep-related breathing disorders, affecting a substantial portion of the adult population and imposing serious health risks. Estimates suggest that nearly one billion individuals worldwide may have OSA when using broad diagnostic criteria, and prevalence in some populations exceeds 50% [1]. Beyond its high prevalence, untreated OSA is associated with excessive daytime sleepiness, impaired cognitive performance, reduced quality of life, and an elevated risk of hypertension, arrhythmias, heart failure, and cardiovascular mortality [2].

Clinically, OSA severity is determined using the apnea–hypopnea index (AHI), which quantifies the number of apnea and hypopnea events per hour of sleep. An apnea represents a complete cessation of airflow lasting at least 10 seconds, while a hypopnea is a partial airflow reduction accompanied by oxygen desaturation [3]. Standard thresholds classify AHI values below 5 as normal, 5–15 as mild, 15–30 as moderate, and above 30 as severe [4]. Although widely adopted, the AHI captures only average event frequency and overlooks key dimensions of disease burden, including the timing, duration, clustering, and physiological impact of events, as well as the degree of sleep fragmentation [5]. The clinical expression of OSA further varies with age, sex, and BMI [6], and the condition shares a bidirectional relationship with cardiovascular disease (CVD), with each contributing to the onset and progression of the other [7].

The diagnostic gold standard for OSA is overnight polysomnography (PSG), which simultaneously measures sleep architecture, respiratory events, oxygen saturation, and autonomic function under controlled laboratory conditions. While comprehensive, PSG is resource-intensive, expensive, and logistically demanding, requiring specialized equipment, trained personnel and overnight monitoring. Many individuals with clinically significant OSA remain undiagnosed due to the barriers inherent in the standard diagnostic pathway [2]. As a result, there is a pressing need for scalable, low-complexity screening tools that can be deployed outside conventional sleep laboratories and capture high-risk individuals earlier.

At the same time, the proliferation of wearable health technology devices has opened up opportunities for continuous monitoring of physiological signals in daily life. Modern consumer devices routinely record heart rate, motion, and in some cases, pulse oximetry, offering unprecedented longitudinal data. For example, heart rate variability (HRV), which reflects the autonomic nervous system’s regulation of the heart, has become accessible through many wearable platforms and holds promise as a screening biomarker [8, 9]. Although these devices lack the full sensing capabilities of a sleep laboratory, their ubiquity and data-rich nature make them attractive for early-stage risk detection of sleep-disordered breathing.

Most HRV-based approaches compress complex autonomic dynamics into a limited set of time- or frequency-domain summary metrics, which may fail to capture the full extent of pathological fluctuations induced by OSA. Recent evidence indicates that sleep apnea disrupts heartbeat-interval correlation patterns across multiple temporal scales rather than within a single frequency band or fixed window [10]. To address this, we utilize scale-dependent detrended fluctuation analysis (sDFA) [11], an extension of nonlinear DFA [12, 13] commonly used in HRV research. Unlike conventional DFA, which reduces temporal dynamics to a single scaling exponent, sDFA tracks how correlation properties evolve across scales, offering a richer view of autonomic regulation in OSA. Our recent work has shown that these multiscale measures capture clinically meaningful risk signatures: sDFA provides strong independent hazard ratios for sudden cardiac death [14] and performs well in detecting long QT syndrome [15]. In addition, its further extension, dynamical DFA [16] (DDFA), has recently proven effective for identifying characteristic patterns of congestive heart failure [17]. Earlier, DDFA has also shown promise in sleep stage detection [18]. These findings underscore the broader diagnostic value of advanced DFA methods such as sDFA and motivate their application to sleep apnea screening in the present study.

It has been previously shown that sDFA can detect OSA in the PhysioNet Apnea–ECG dataset [19], which provides an important benchmark for further development. To build on these findings, we extend this evaluation to the first phase of the Sleep Heart Health Study (SHHS1). This is a much larger and more heterogeneous cohort of more than 5,800 participants spanning all apnea severity levels and including individuals with cardiovascular disease. Using this dataset, we evaluate mild, moderate, and severe apnea and perform subgroup analyses stratified by cardiovascular disease (CVD) status. Our goal is to determine whether incorporating scale information improves apnea detection across these clinically relevant groups and to explore the utility of sDFA as a more accessible, early stage screening tool for sleep apnea outside the sleep laboratory.

## Methods

### Data and preprocessing

We analyzed 5,804 overnight PSG recordings from SHHS1, obtained through the National Sleep Research Resource [20, 21]. Each recording is accompanied by 1,271 descriptive variables, spanning from demographic details such as age, sex, and BMI to clinical data on electrocardiograms (ECG), CVD, and OSA.

**Fig. 1:**
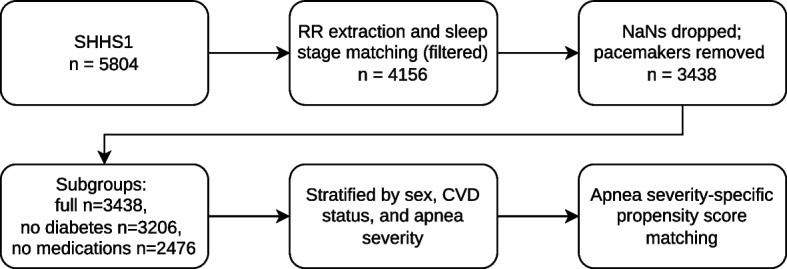
Patient selection pipeline for the analysis

RR intervals (RRI) were extracted from ECG signals using a delineation algorithm [22]. The SHHS1 dataset includes sleep stage annotations in 30 s epochs, which we align with the extracted RR intervals. This alignment enables the removal of extended periods of wakefulness at the beginning of the PSG recordings, while preserving nighttime awakenings that may be related to OSA. A rolling median filter (51-beat window) was applied to the RRI series. Beats with RRI outside (0.85*n*, 1.15*n*), where *n* is the local median, were removed. Participants with less than 80% valid RRIs were excluded from the analysis. Participants with pacemakers ($n = 29$) were excluded from the analysis, as were those with missing metadata values. The resulting dataset comprised RRI data from 3,438 subjects, including 1,519 males and 1,919 females. The patient selection flowchart is shown in Fig. 1. The full dataset was used for the main analyses, while smaller subgroups excluding participants with diabetes ($n = 3206$) and those using relevant medications ($n = 2476$) were analyzed in supplementary sensitivity analyses [see Additional File 1].

Sleep apnea severity in this study is defined using the AHI, which quantifies the number of apnea and hypopnea events per hour of sleep. An apnea corresponds to a complete cessation of airflow for at least 10 seconds, while a hypopnea reflects a partial reduction in airflow accompanied by oxygen desaturation [3]. Following established clinical thresholds, we classify subjects as healthy (AHI < 5), mild OSA (5–15), moderate OSA (15–30), or severe OSA (AHI > 30) [4], solely based on AHI score. Although the AHI does not capture factors such as event duration, timing, or sleep fragmentation [5], it remains the standard measure for defining severity categories and is therefore used here for group assignment. We use the AHI score (*nsrr_ahi_hp4u_aasm15*) harmonized by the National Sleep Research Resource team, which is defined as All apneas + hypopneas with $\ge 30$% nasal cannula [or alternative sensor] reduction with $\ge 4$% oxygen desaturation)/hour of sleep [20, 21].

Because OSA severity is influenced by demographic and physiological characteristics, such as age, sex, and BMI [6], and because OSA and CVD are closely linked [7], we explicitly account for these factors in our analysis. The dataset is first divided into four severity groups based on AHI (healthy, mild, moderate, severe), and each severity group is further split according to CVD status (’–’ for no CVD, ’+’ for CVD). The dataset defines cardiovascular disease as the presence of congestive heart failure, myocardial infarction, or angina pectoris; therefore, participants with arrhythmias or atrial fibrillation were not classified as having CVD and were included in the analysis.

To reduce confounding, the data were stratified by sex, and propensity score matching [23] was used to identify age- and BMI-matched individuals from the healthy group for each apnea severity group. Propensity scores were estimated via logistic regression using age and BMI as covariates, followed by nearest-neighbor matching on the logit of the propensity score. All apnea cases were retained, and no subsampling of the treated group was performed to ensure stable matching, particularly in smaller strata.

### Time-series analysis of beat-to-beat intervals

In this study, we detect the presence and severity of sleep apnea using both conventional heart rate variability (cHRV) metrics [9] and advanced multiscale measures of heart rate variability [11]. We compute several established cHRV indices that are widely used in sleep and cardiovascular research. In the time domain, we use the root mean square of successive differences (RMSSD), which reflects short-term parasympathetic modulation. In the frequency domain, we evaluate power in the low-frequency (LF; 0.04–0.15 Hz) and high-frequency (HF; 0.15–0.40 Hz) bands, along with their ratio (LF/HF), an indicator of sympathovagal balance. We further include the nonlinear Poincaré plot ratio SD1/SD2 and the mean RR interval as complementary descriptive features.

To capture correlation properties that extend beyond these summary metrics, we employ DFA. It is a well-established method for quantifying long-range temporal correlations in nonstationary physiological time series  [12, 13, 24, 25]. DFA characterizes how fluctuations in the integrated RR-interval signal grow with scale, yielding a scaling exponent $\alpha$. Traditional DFA analysis typically distinguishes short-term ($\alpha _1$, 4–16 beats) and long-term ($\alpha _2$, 16–64 beats) exponents, which have been linked to baroreflex mechanisms and slower autonomic regulation, respectively [9].

sDFA [11] extends standard DFA by estimating a continuous scaling spectrum ($\alpha (s)$) across a wide range of overlapping temporal scales. Instead of restricting the analysis to two predefined windows, sDFA reveals how correlation properties evolve gradually from short to long time scales, providing a more detailed view of autonomic dynamics [14, 15, 17] In this study, we apply second-order sDFA over scales from 4 to 1000 beats. The interpretation of $\alpha$ values across different regimes is provided in Table 1.

**Table 1:** Interpretation of DFA scaling exponents $\alpha$ [16]

Scaling exponent	Interpretation
$0< \alpha < 0.5$	anti-correlated
$\alpha = 0.5$	white noise
$0.5< \alpha < 1$	correlated
$\alpha = 1$	1/*f* noise
$1< \alpha < 1.5$	anti-correlated increments
$\alpha = 1.5$	Brownian noise
$1.5< \alpha < 2$	correlated increments

### Area under the receiver operating characteristic curve

The receiver operating characteristic (ROC) curve and its corresponding area under the curve (AUC) were used to asses the performance of each HRV measure to distinguish between apnea and non-apnea subjects. This metric is widely regarded as a robust measure for evaluating diagnostic and classification models, particularly in physiological and biomedical contexts [26, 27].

Each HRV measure was treated as a continuous physiological marker of apnea and evaluated independently. The HRV values were used directly as continuous, non-thresholded scores in the ROC analysis, without training or applying a classifier. ROC curves were generated by sweeping a decision threshold across the full range of each metric, allowing assessment of how well the marker alone ranked apnea subjects relative to non-apnea subjects. Accordingly, the AUC quantifies the probability that a randomly selected apnea subject exhibits a more extreme HRV value than a randomly selected healthy subject, reflecting discrimination based solely on ranking rather than on a fixed decision rule. To maintain consistency across predictors, AUC values below 0.5 were inverted ($\mathrm {AUC^{\prime }} = 1-\textrm{AUC}$), ensuring that higher values uniformly indicated stronger discriminative ability, irrespective of the direction of association.

### Power analysis

To estimate the statistical power of the comparisons between the apnea and non-apnea groups, we performed a post-hoc power analysis. For each condition (sex $\times$ apnea severity $\times$ cardiovascular-disease status), we selected the scale that yielded the highest AUC value. Within each matched dataset, we extracted the corresponding scaling exponent $\alpha$ values for subjects with and without sleep apnea and calculated the mean $(\bar{X}_{\text {healthy}}, \bar{X}_\text {apnea})$ and standard deviation $(s_\text {healthy}^2, s_\text {apnea}^2)$ in each group. Cohen’s *d* was computed as the standardized mean difference between groups using the pooled standard deviation

$$\begin{aligned} & d = \frac{\bar{X}_{\text {healthy}} - \bar{X}_{\text {apnea}}}{s_p},\ & s_p = \sqrt{\frac{(n_1 - 1)s_\text {healthy}^2 + (n_2 - 1)s_\text {apnea}^2}{n_1 + n_2 - 2}}, \end{aligned}$$

where $n_1 = n_2 = n$ due to pairwise propensity-score matching. Statistical power for a two-sample *t*-test (two-tailed, 0.05 confidence interval) was then estimated from the observed effect size. The resulting power values quantify the probability of detecting the observed effect at the given sample size [28].

## Results

Tables 2, 3 and 4 present the matched cohorts used in the subsequent analyses. Subjects with CVD were significantly older than those without CVD across all sex and apnea severity strata (all $p < 0.01$, Welch’s t-test).

**Table 2:** Matched subjects with mild sleep apnea: age and BMI (mean ± standard deviation), subject count, sex, and cardiovascular disease (CVD) status

Apnea	AHI	Age (y)	BMI (kg/m^2^)	*N*	Sex	CVD
healthy	2.3 ± 1.4	60.8 ± 10.0	27.6 ± 3.7	379	Male	-
mild	9.2 ± 3.0	62.1 ± 9.7	28.5 ± 3.8	379	Male	-
healthy	2.4 ± 1.4	70.4 ± 9.2	28.6 ± 4.1	144	Male	+
mild	9.8 ± 2.9	67.5 ± 9.3	27.3 ± 3.3	144	Male	+
healthy	2.0 ± 1.3	64.1 ± 9.1	29.4 ± 4.8	409	Female	-
mild	8.9 ± 2.8	63.9 ± 10.2	29.5 ± 5.7	409	Female	-
healthy	2.4 ± 1.4	72.4 ± 7.4	28.3 ± 4.6	112	Female	+
mild	8.8 ± 2.6	73.5 ± 8.7	28.6 ± 5.1	112	Female	+

**Table 3:** Matched subjects with moderate sleep apnea: age and BMI (mean ± standard deviation), subject count, sex, and CVD status

Apnea	AHI	Age (y)	BMI (kg/m^2^)	*N*	Sex	CVD
healthy	2.4 ± 1.5	64.7 ± 9.1	28.4 ± 4.2	195	Male	-
moderate	21.1 ± 4.3	64.8 ± 9.9	29.3 ± 4.4	195	Male	-
healthy	2.5 ± 1.4	68.3 ± 7.3	28.2 ± 3.0	86	Male	+
moderate	21.2 ± 4.3	70.2 ± 9.3	29.6 ± 4.1	86	Male	+
healthy	2.1 ± 1.4	65.1 ± 7.8	30.5 ± 5.7	145	Female	-
moderate	20.5 ± 4.1	65.9 ± 10.1	30.3 ± 6.4	145	Female	-
healthy	2.2 ± 1.5	70.0 ± 8.6	30.7 ± 4.3	32	Female	+
moderate	20.4 ± 4.2	72.2 ± 9.5	31.1 ± 6.4	32	Female	+

**Table 4:** Matched subjects with severe sleep apnea: age and BMI (mean ± standard deviation), subject count and CVD status

Apnea	AHI	Age (y)	BMI (kg/m^2^)	*N*	Sex	CVD
healthy	2.7 ± 1.4	60.7 ± 7.7	29.9 ± 4.1	101	Male	-
severe	46.9 ± 14.2	63.1 ± 9.2	31.1 ± 4.6	101	Male	-
healthy	2.5 ± 1.5	65.6 ± 7.5	29.0 ± 3.2	47	Male	+
severe	47.5 ± 15.7	69.6 ± 10.6	30.6 ± 5.1	47	Male	+
healthy	2.3 ± 1.4	66.1 ± 8.6	31.9 ± 5.7	62	Female	-
severe	47.1 ± 15.8	66.8 ± 9.2	32.3 ± 6.7	62	Female	-
healthy	2.6 ± 1.3	75.2 ± 3.7	28.3 ± 5.1	13	Female	+
severe	45.4 ± 22.8	78.5 ± 9.5	29.1 ± 9.0	13	Female	+

**Fig. 2:**
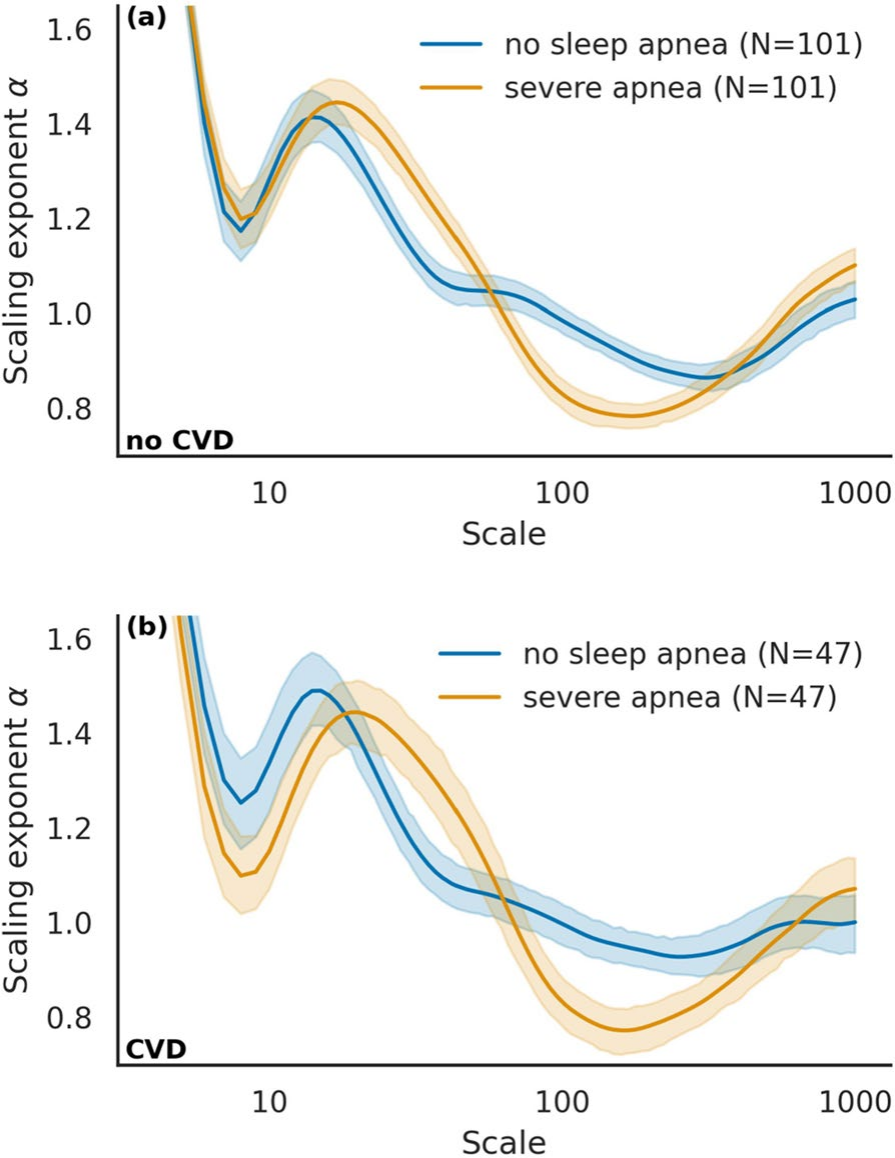
Scale-dependent sDFA exponent in severe apnea and healthy male participants. sDFA scaling exponent ($\alpha$) as a function of scale for severe apnea and healthy male participants without (**a**) and with (**b**) cardiovascular disease (CVD). For both groups (no CVD: $n=101$; CVD: $n=47$), $\alpha$ decreases at short scales ($s \le 8$), reaches a local maximum around scales 20–25, and then gradually declines before rising again at very large scales ($s \ge 200$). Severe apnea patients consistently show lower $\alpha$ values and a steeper decline than healthy individuals, whose curves display a milder, saddle-like pattern around scales 50–100. Overall trajectories are similar in the no CVD and CVD groups, although the CVD subgroup exhibits a lower short-scale minimum

We compared the sDFA scaling exponents $\alpha (s)$ between participants with and without sleep apnea. Figure 2a shows $\alpha (s)$ with 95% confidence intervals for male participants with severe apnea and no CVD, and Fig. 2b presents the corresponding results for those with CVD.

Across both groups, $\alpha (s)$ followed a characteristic pattern: it decreased at short scales ($s \le 8$) to 1.2, rose to a local maximum of 1.45–1.5 around scales 20–25, and then gradually declined to 0.8–1.0 before increasing again at very large scales ($s \ge 200$). Severe apnea participants with CVD [Fig. 2b] showed lower short-scale $\alpha$ values than those without CVD [Fig. 2a], although the overall trajectories were similar. The decline was consistently steeper in participants with severe apnea, whereas those without sleep apnea exhibited a milder, saddle-like pattern around scales 50–100, with $\alpha (s)$ remaining higher at 1.0 and never reaching the low levels of 0.8 observed in severe apnea. These patterns were consistent across both sexes and across different apnea severity levels.

**Fig. 3:**
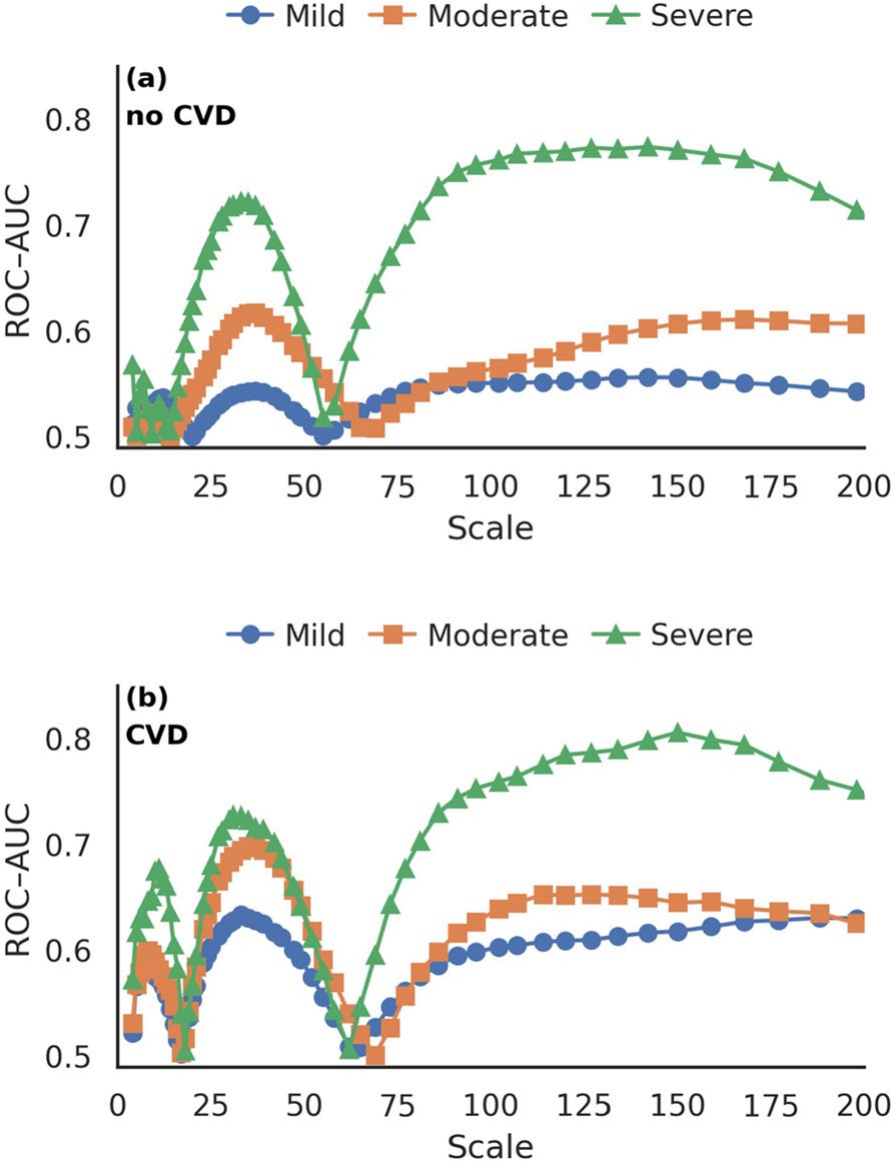
Scale-dependent AUC of sDFA across apnea severity and CVD status. Area under the receiver operating characteristic curve (AUC) of the sDFA scaling exponent ($\alpha$) as a function of scale for male participants without (**a**) and with (**b**) CVD, shown separately for all apnea severity groups. In both groups, two principal AUC peaks are observed: an initial local maximum of approximately 0.72 at intermediate scales ($s \approx 30$–45), followed by a decline toward chance level (AUC $\approx 0.5$) at scales 50–60 and a secondary increase to approximately 0.75 at scales 60–100. Beyond $s \approx 100$, AUC values remain relatively stable around 0.7 before decreasing again at very large scales ($s \ge 200$). In the CVD subgroup, short-scale AUC values ($s \le 20$) exceed 0.65 for all apnea severity groups, with the mild and moderate groups showing short-scale AUCs comparable to those of the severe group; this convergence is not observed in participants without CVD, where short-scale AUCs for mild and moderate apnea remain clearly lower than those for severe apnea. Participants with CVD also exhibit a distinct saddle-like increase in AUC in the severe apnea group

Next we consider the AUC values of the scaling exponent $\alpha$ across all scales. Figure 3 shows the discriminative performance for male participants without (a) and with (b) cardiovascular disease (CVD), separated by apnea severity group. The overall pattern of AUC values is similar without and with comorbid CVD. Across all apnea severity groups, two main peaks are observed: the first around scales 30–45, where the AUC reaches a local maximum of 0.72, followed by a decline to 0.5 at scales 50-60 and a secondary rise to 0.75 between scales 60–100. Beyond scale 100, the AUC remains relatively stable at 0.7 before decreasing again at very large scales ($s \ge 200$).

Notably, the severe apnea group with CVD shows a distinct saddle-like increase in AUC not present in the corresponding no-CVD group. In addition, the local maxima at short scales ($s \le 20$) are higher for all apnea groups when CVD is present (> 0.65), and the mild and moderate groups also exhibit higher peaks around scales 30–45. Similar trajectories are observed in the female subgroups, but due to the size of the data especially for the CVD groups, the results do not have the same statistical power as for the male subgroups.

In order to statistically test the reliability of our study, we performed a power analysis for all groups as described in Power analysis sections. The results are shown in Table 5 alongside the best AUC scores and corresponding scales.

**Table 5:** Scales with the highest AUC scores for each apnea type and cardiovascular condition, alongside their statistical power

Condition	Scale	AUC	*N*	Sex	CVD	Power
mild apnea	142	0.56	379	Male	-	0.793
moderate apnea	37	0.62	195	Male	-	0.990
severe apnea	142	0.77	101	Male	-	1.000
mild apnea	33	0.63	144	Male	+	0.960
moderate apnea	35	0.70	86	Male	+	0.999
severe apnea	150	0.81	47	Male	+	1.000
mild apnea	210	0.58	409	Female	-	0.953
moderate apnea	142	0.64	145	Female	-	0.995
severe apnea	168	0.82	62	Female	-	1.000
mild apnea	49	0.61	112	Female	+	0.820
moderate apnea	28	0.65	32	Female	+	0.523
severe apnea	188	0.79	13	Female	+	0.670

All groups exceeded the conventional 0.8 power threshold, except mild apnea without CVD (males), moderate apnea with CVD (females), and severe apnea with CVD (females)

**Fig. 4:**
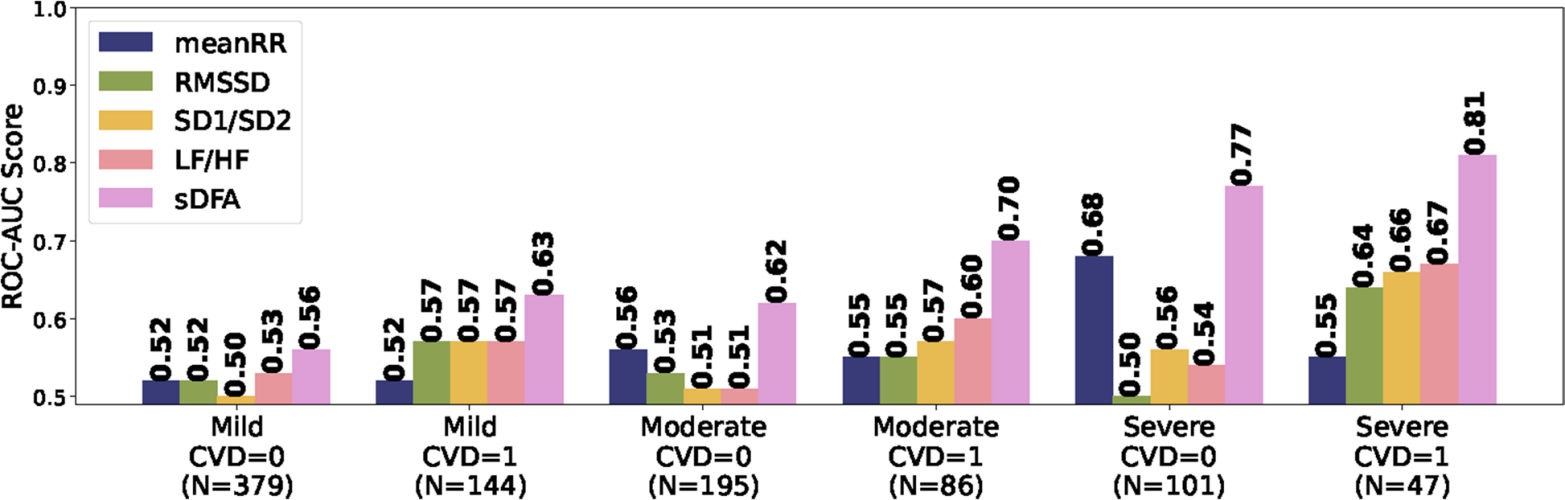
Discriminative performance of sDFA and HRV metrics across apnea severity and CVD. AUC scores for sDFA and conventional HRV (cHRV) metrics across apnea severity levels and CVD status in male participants. Across all subgroups, sDFA achieves the highest discriminative performance, with AUC values ranging from 0.56 to 0.81 and a maximum AUC of 0.81 observed in the severe apnea group. In contrast, no conventional HRV metric consistently emerges as the second-best performer, with performance varying across severity and CVD subgroups. Notably, root mean square of successive differences (RMSSD) shows relatively strong performance in participants with severe apnea and CVD, reaching an AUC of approximately 0.69

All the groups pass the conventional 0.8 power threshold, with the exception of the mild apnea without CVD male subgroup, moderate apnea with CVD female subgroup and severe apnea with CVD female subgroup. The table also reports the scale corresponding to the highest AUC score for each subgroup. To compare sDFA with cHRV measures, AUC scores were computed in the same manner for the other metrics, and the results are presented in Fig. 4.

As shown in Fig. 4, sDFA outperforms cHRV methods with a ROC-AUC scores ranging from 0.56 to 0,81, particularly in the severe apnea group (0.81). No single metric consistently emerges as the second-best performer, as the cHRV measures show no clear or consistent pattern across subgroups. Interestingly, RMSSD performs unexpectedly well among subjects with severe apnea and CVD (ROC-AUC of 0.69). In smaller strata (e.g., severe apnea with CVD), performance estimates were more sensitive to matching configuration, whereas larger strata yielded stable ROC–AUC values.

Although the primary analyses focus on male participants, qualitatively similar patterns were observed in the female subgroups. Detailed results for female participants across apnea severity and cardiovascular disease subgroups, as well as for male participants with mild and moderate apnea, are provided in the additional file [see Additional file 1].

In addition, the potential influence of diabetes and medication use was also evaluated separately. Detailed analyses excluding participants with diabetes ($n_{dia} = 232$) and those taking angiotensin-converting enzyme (ACE) inhibitors, beta-blockers, or calcium-channel blockers (CCIR, CCBSR, CCBT), stratified by sex and CVD status ($n_{med} = 962$), are presented in additional file [see Additional File 1]. Across these sensitivity analyses, sDFA continued to consistently outperformed cHRV metrics. However, the reduced sample sizes, especially in the case of no medications, warrant cautious interpretation of these findings.

## Discussion

This study demonstrates that sDFA provides a more reliable and informative marker for sleep apnea than conventional HRV metrics in a cohort of 3,438 community participants selected from the Sleep Heart Health Study. Across all male and female subgroups, sDFA consistently achieved higher AUC values than mean RR, RMSSD, LF/HF, and SD1/SD2. sDFA was especially effective in detecting severe apnea, where conventional metrics showed inconsistent performance with no clear second-best alternative. These findings indicate that incorporating scale information captures physiologically meaningful fluctuations linked to apnea severity.

The improved performance of sDFA likely arises from its sensitivity to multi-scale autonomic dysregulation. Sleep apnea induces repeated cycles of hypoxia, arousal, and cardiorespiratory instability, which unfold over a range of temporal scales [29]. Conventional time- and frequency-domain HRV measures compress this complex structure into fixed windows or spectral bands, potentially obscuring distinctive apnea-related signatures. In contrast, sDFA quantifies how correlations in the RRI time series evolve across scales, effectively mapping where pathological dynamics emerge. The pronounced peaks at scales 30–45 and 60–100 suggest these windows may reflect characteristic interactions between autonomic recovery times, respiratory oscillations, and sympathetic bursts that accompany apnea events.

The results highlight several clinically relevant advantages. First, sDFA operates solely on RRI time series and is therefore not limited to ECG-derived RRI. While this study uses ECG, previous work has demonstrated that sDFA can also be applied to intervals extracted from photoplethysmography (PPG) signals [18], supporting its compatibility with a wide range of wearable and home-monitoring technologies. Although PPG signal quality can vary across individuals and may be influenced by factors such as skin tone and sensor characteristics [30], the proposed framework itself remains modality-agnostic and depends primarily on the accuracy of inter-beat interval estimation. Second, identifying optimal diagnostic scales could inform simplified algorithms that compute only a subset of scales, reducing computational overhead for real-time or embedded systems. Finally, because sDFA captures broader autonomic dysregulation rather than only respiratory-driven variability, it may be particularly useful in patients with comorbidities, such as the case with cardiovascular diseases, where cHRV metrics often lose discriminative power. Thus, sDFA could serve as a complementary or alternative feature in automated sleep apnea screening frameworks.

## Strengths and Limitations

A major strength of this work is the systematic evaluation of sDFA across scales and subgroups, revealing patterns that would be obscured by a single-slope DFA approach. Additionally, comparing performance across several cHRV metrics ensures that the advantage of sDFA is not metric-specific but robust. Nonetheless, the analysis has several limitations. As HRV-based measures, including DFA and sDFA, primarily reflect autonomic regulation rather than respiration itself, they provide an indirect assessment of apnea and may be influenced by factors other than breathing disturbances, such as stress, arousals, comorbidities, and medication use. Female CVD subgroups were relatively small, which may reduce the stability of performance estimates. In such smaller strata, limited covariate overlap between groups can increase sensitivity to the matching configuration; therefore, these subgroup-specific results should be interpreted with caution. The cross-sectional nature of the study prevents insights into night-to-night variability or progression. Furthermore, sleep apnea severity was defined using the AHI, a nightly summary measure that does not preserve the temporal distribution or duration of respiratory events. As a result, direct alignment between individual apnea events and transient HRV/sDFA dynamics could not be assessed. Data quality and the accuracy of gold-standard annotations may also introduce uncertainty. Finally, although sDFA offers interpretability at the scale level, the physiological meaning of individual scales requires further investigation.

Future work should examine how various cardiovascular diseases, such as myocardial infarction, congestive heart failure, or angina, modulate the scale-dependent structure of HRV. In this study, we observed subgroup-specific patterns (e.g., the saddle-like increase in severe apnea with CVD), suggesting that distinct pathophysiological mechanisms may leave characteristic multi-scale signatures. Examining more specific CVD classifications could reveal whether particular conditions disproportionately affect short- versus long-term correlations and help establish CVD-specific “scale fingerprints”. In parallel, future studies should assess whether sDFA retains its discriminative power in ambulatory recordings and under varying sampling frequencies or artifact conditions. In addition, sleep-stage–stratified and event-aligned analyses could be incorporated to disentangle apnea-specific autonomic alterations from stage-dependent HRV dynamics. Integrating sDFA with machine-learning frameworks and additional physiological signals (e.g., respiration or oxygen saturation) may further improve performance and clarify how specific scales relate to respiratory timing, arousal dynamics, or autonomic reflex latency. Ultimately, validating sDFA in larger, multi-center cohorts will be essential for establishing it as a robust and clinically meaningful biomarker for detecting sleep apnea.

## Conclusions

In this study, we evaluated sDFA as an HRV-based approach for detecting sleep apnea in a large clinical cohort. By analyzing 3,438 participants and stratifying the data by apnea severity and cardiovascular disease status, we showed that sDFA consistently outperforms conventional HRV metrics and reveals multiscale autonomic signatures that are not captured by standard time- or frequency-domain measures. Importantly, sensitivity analyses excluding participants with diabetes and those using relevant cardiovascular medications yielded comparable results, suggesting that the observed patterns are robust in the presence of these common clinical confounders. Together, these findings demonstrate the value of scale-resolved analysis for characterizing how sleep apnea alters cardiac dynamics and highlight the limitations of relying solely on single-metric HRV approaches.

The ability of sDFA to detect meaningful changes in severe apnea and to maintain discriminative performance even in individuals with comorbid CVD suggests that it may serve as a robust and clinically relevant biomarker. Because sDFA operates directly on RR-interval time series, the method is not limited to ECG-based recordings; future work should explore its application to PPG signals commonly available in consumer wearables. Combining sDFA with machine-learning frameworks and additional physiological signals such as respiration or oxygen saturation may further enhance performance and support the development of richer multimodal screening tools. These extensions could substantially improve accessibility by enabling large-scale, efficient, and accurate screening in real-world settings. Continued clinical validation and a deeper investigation of CVD-specific scale signatures will be important for refining sDFA as a practical tool for early detection of sleep apnea.

## Supplementary Information

Supplemetary Material 1.

## Data Availability

The data used in this study are from the Sleep Heart Health Study (SHHS) and were obtained through the National Sleep Research Resource (NSRR). SHHS data are available to qualified researchers via NSRR, subject to approval of a data use agreement.

## Abbreviations

AHI: Apnea–hypopnea index
BMI: Body mass index
CVD: Cardiovascular disease
HRV: Heart rate variability
OSA: Obstructive sleep apnea
PPG: Photoplethysmography
RRI: R–R interval
sDFA: Scale-dependent detrended fluctuation analysis
