# Supplementary material for: Detecting sleep apnea using non-linear measures of heart rate variability
Source: Respir Res. 2026 Mar 19;27:185. doi: 10.1186/s12931-026-03621-6 (PMC13122967; doi:10.1186/s12931-026-03621-6)
Supplement: Supplementary file 1 — Supplemetary Material 1. [file 12931_2026_3621_MOESM1_ESM.pdf]

Additional file 1

Supplement to: Detecting sleep apnea using non-linear measures of heart rate variability

S1 Results for male participants

Figures S1 and S2 show the scaling exponent (α) as a function of scale in male subjects with mild and moderate apnea, respectively.

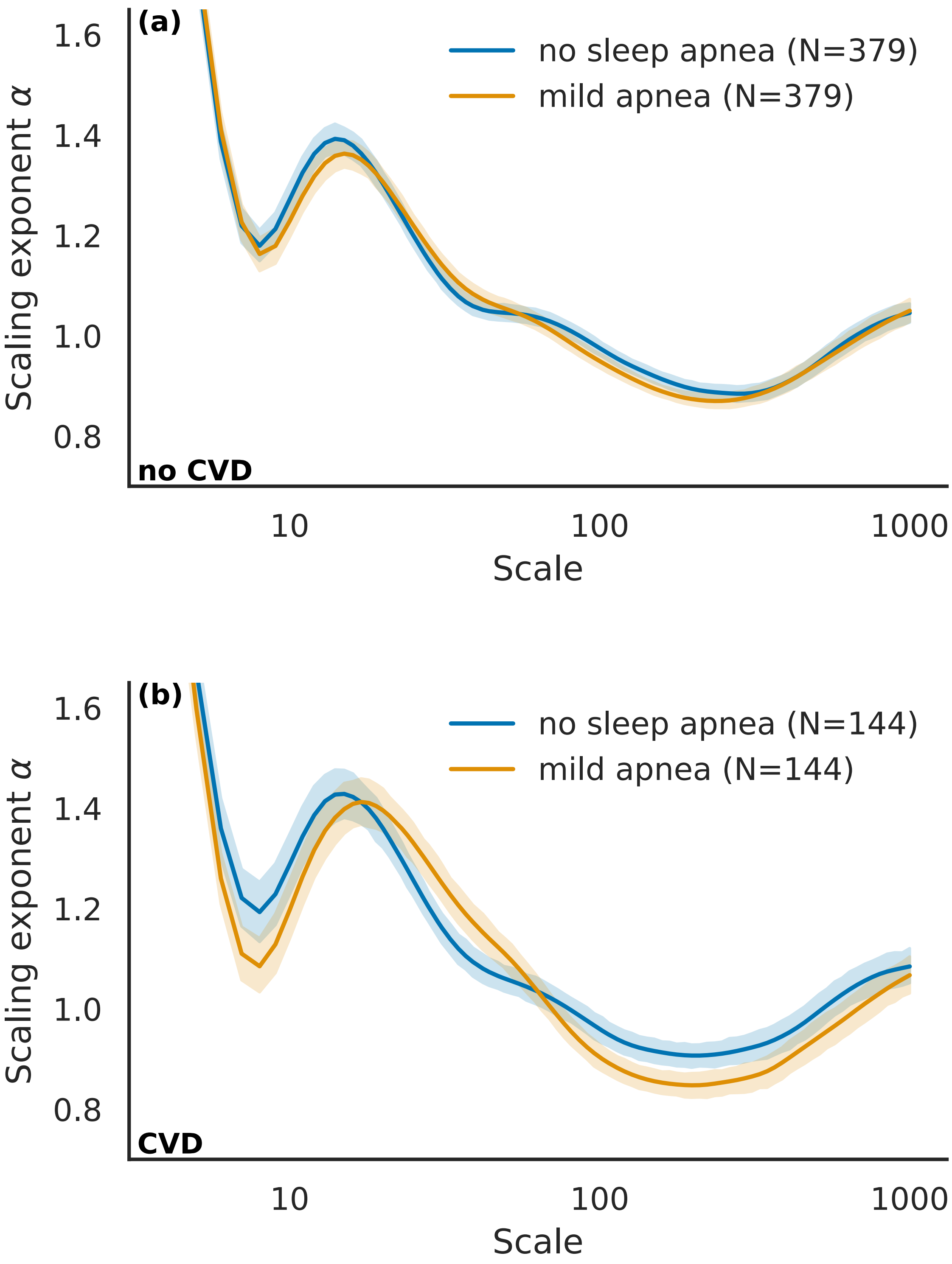

Figure S1: Scale-dependent DFA exponent (α) as a function of scale in male subjects with mild apnea and healthy controls, shown separately for those without (a) and with (b) cardiovascular disease (CVD).

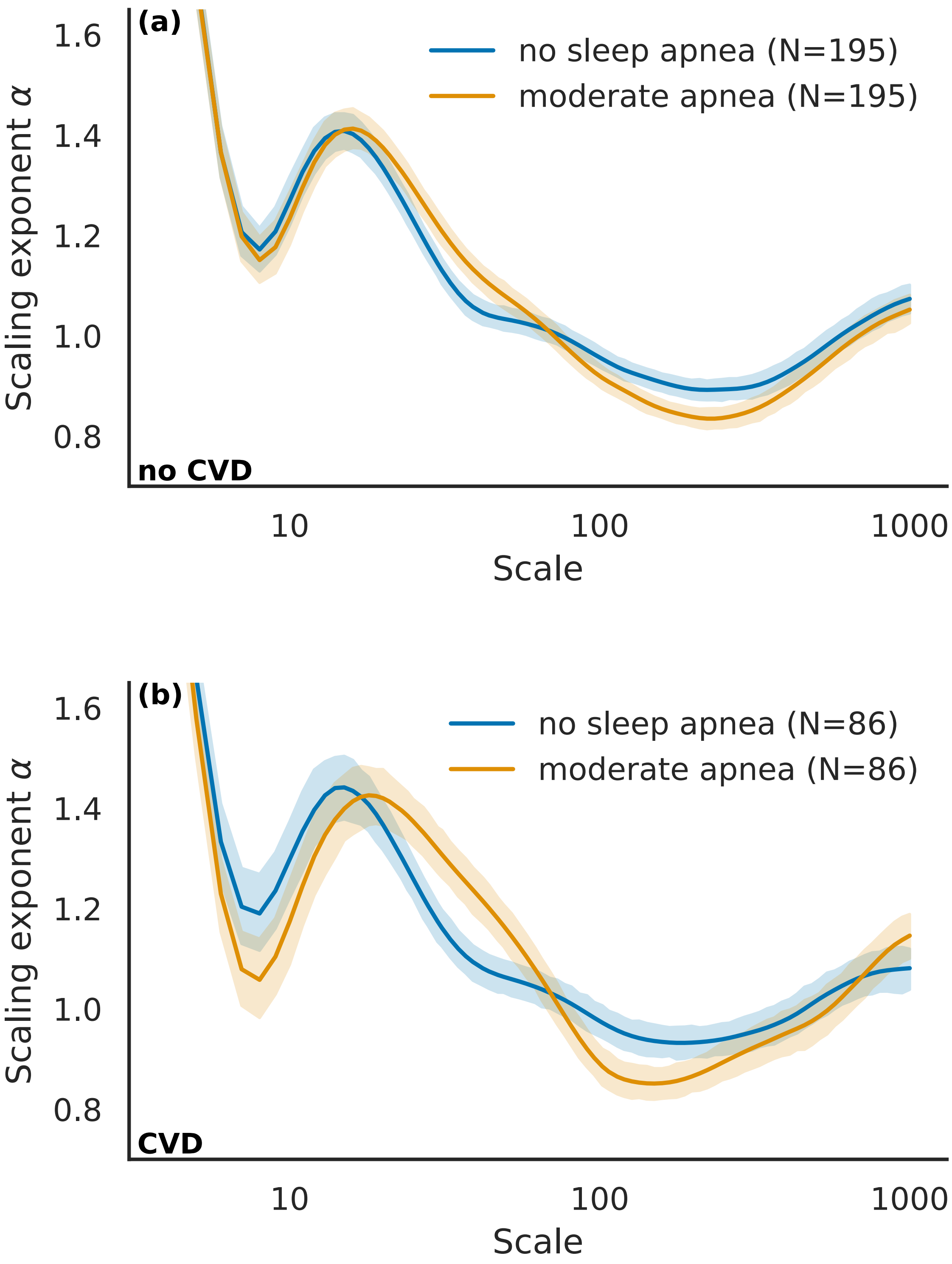

Figure S2: Scale-dependent DFA exponent (α) as a function of scale in male subjects with moderate apnea and healthy controls, shown separately for those without (a) and with (b) cardiovascular disease (CVD).

S2 Results for female participants

Figures S3, S4, and S5 show the scaling exponent (α) as a function of scale in female subjects with mild, moderate, and severe apnea, respectively. The corresponding ROC–AUC values are presented in Fig. S6 and compared with conventional HRV metrics in Fig. S7.

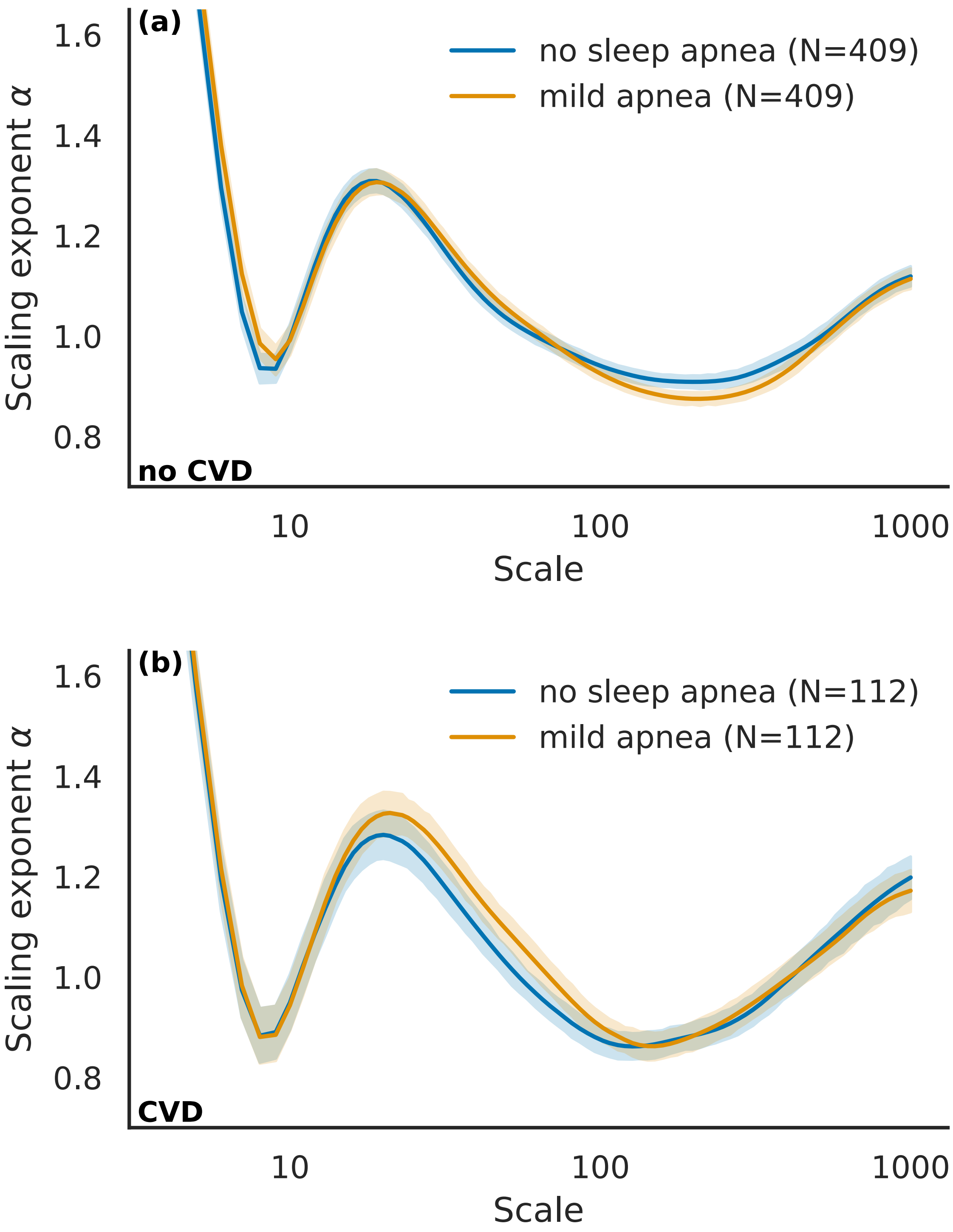

Figure S3: Scale-dependent DFA exponent (α) as a function of scale in female subjects with mild apnea and healthy controls, shown separately for those without (a) and with (b) cardiovascular disease (CVD).

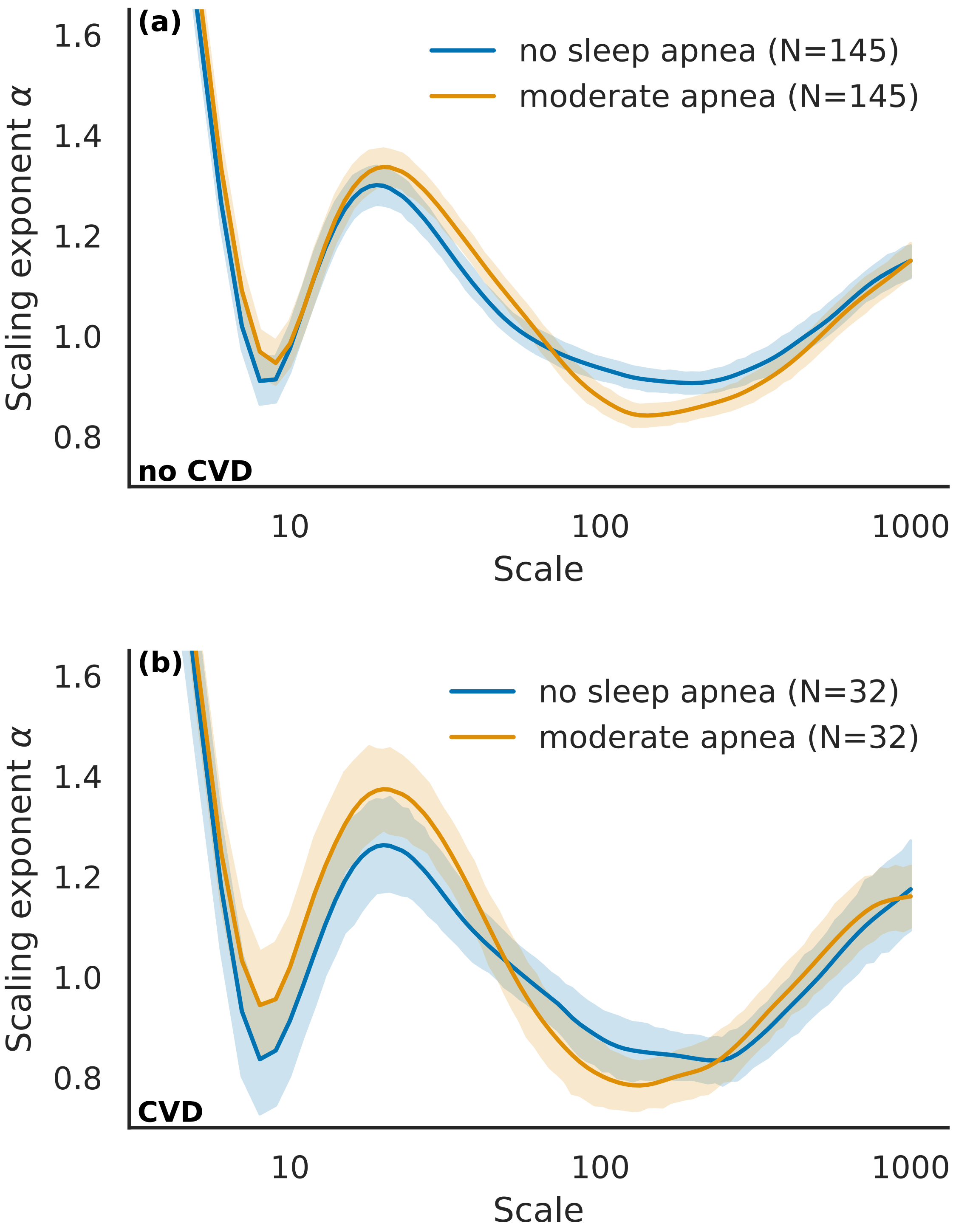

Figure S4: Scale-dependent DFA exponent (α) as a function of scale in female subjects with moderate apnea and healthy controls, shown separately for those without (a) and with (b) cardiovascular disease (CVD).

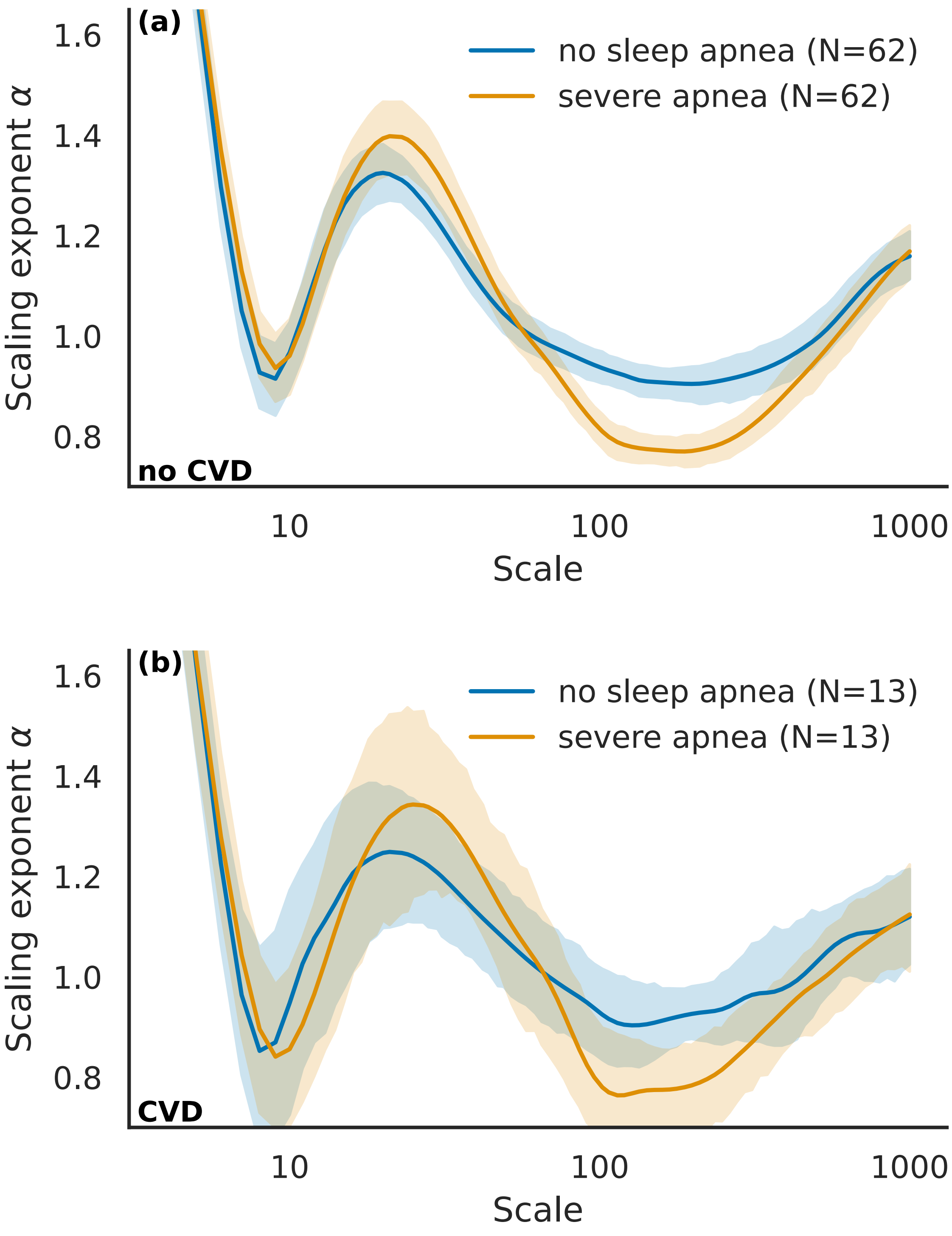

Figure S5: Scale-dependent DFA exponent (α) as a function of scale in female subjects with severe apnea and healthy controls, shown separately for those without (a) and with (b) cardiovascular disease (CVD).

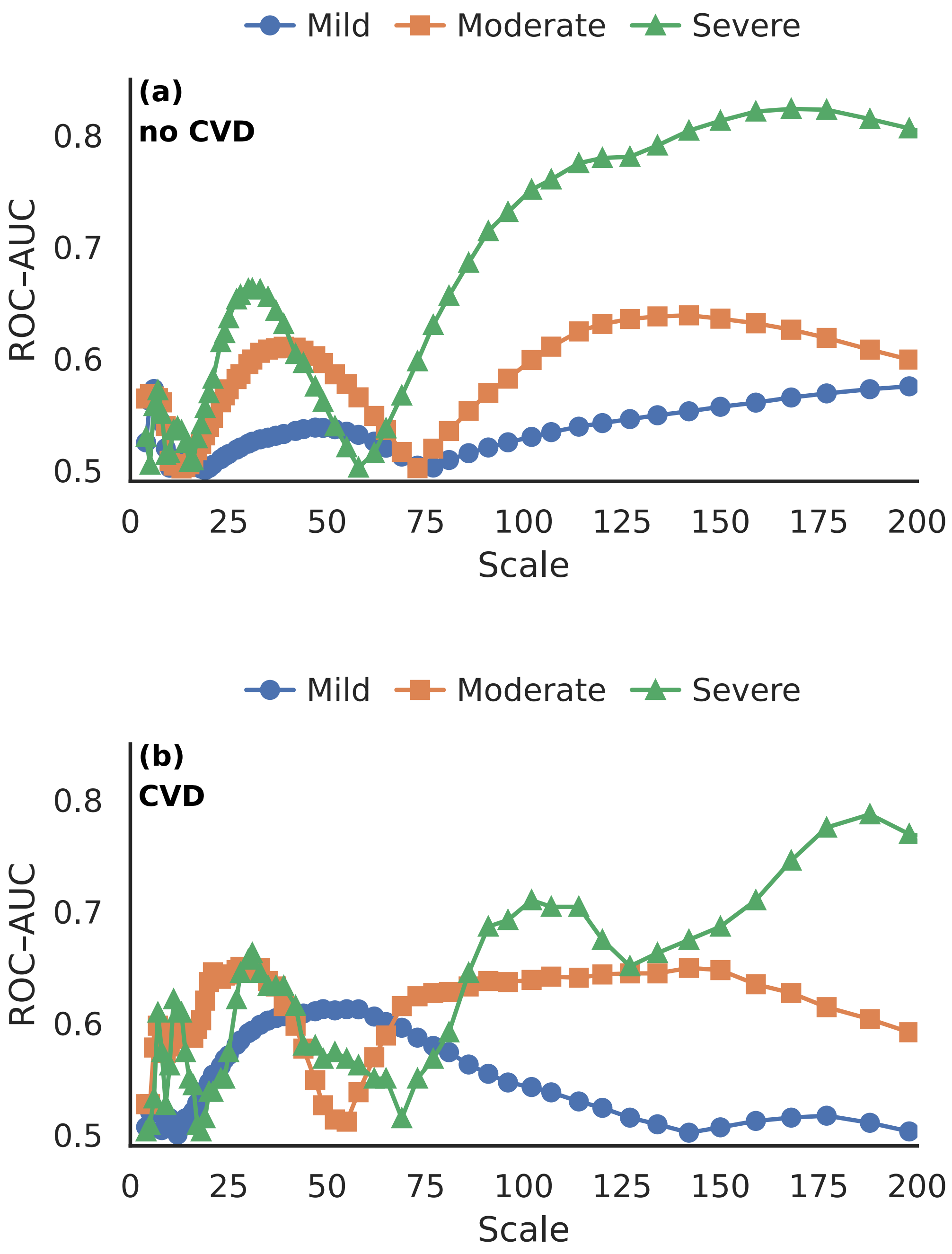

Figure S6: AUC of the scaling exponent (α) as a function of scale in female participants without (a) and with (b) cardiovascular disease (CVD), across all apnea severity levels.

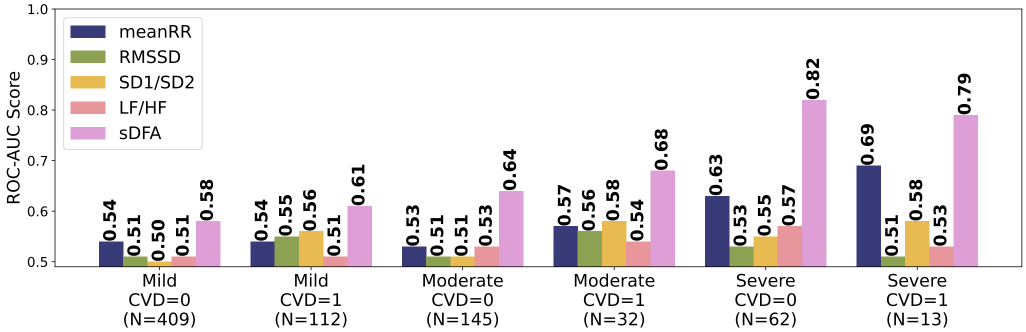

Figure S7: AUC scores of different HRV methods across apnea severity levels and CVD status in female subjects. Consistent with the results in males, performance varies between subgroups, while sDFA outperforms conventional HRV metrics across all groups.

S3 Results for male participants without diabetes

Figures S8, S9, and S10 show the scaling exponent (α) as a function of scale in male subjects with mild, moderate, and severe apnea, respectively. The corresponding ROC–AUC values are presented in Fig. S11 and compared with conventional HRV metrics in Fig. S12. Subjects with diabetes were excluded from the analysis.

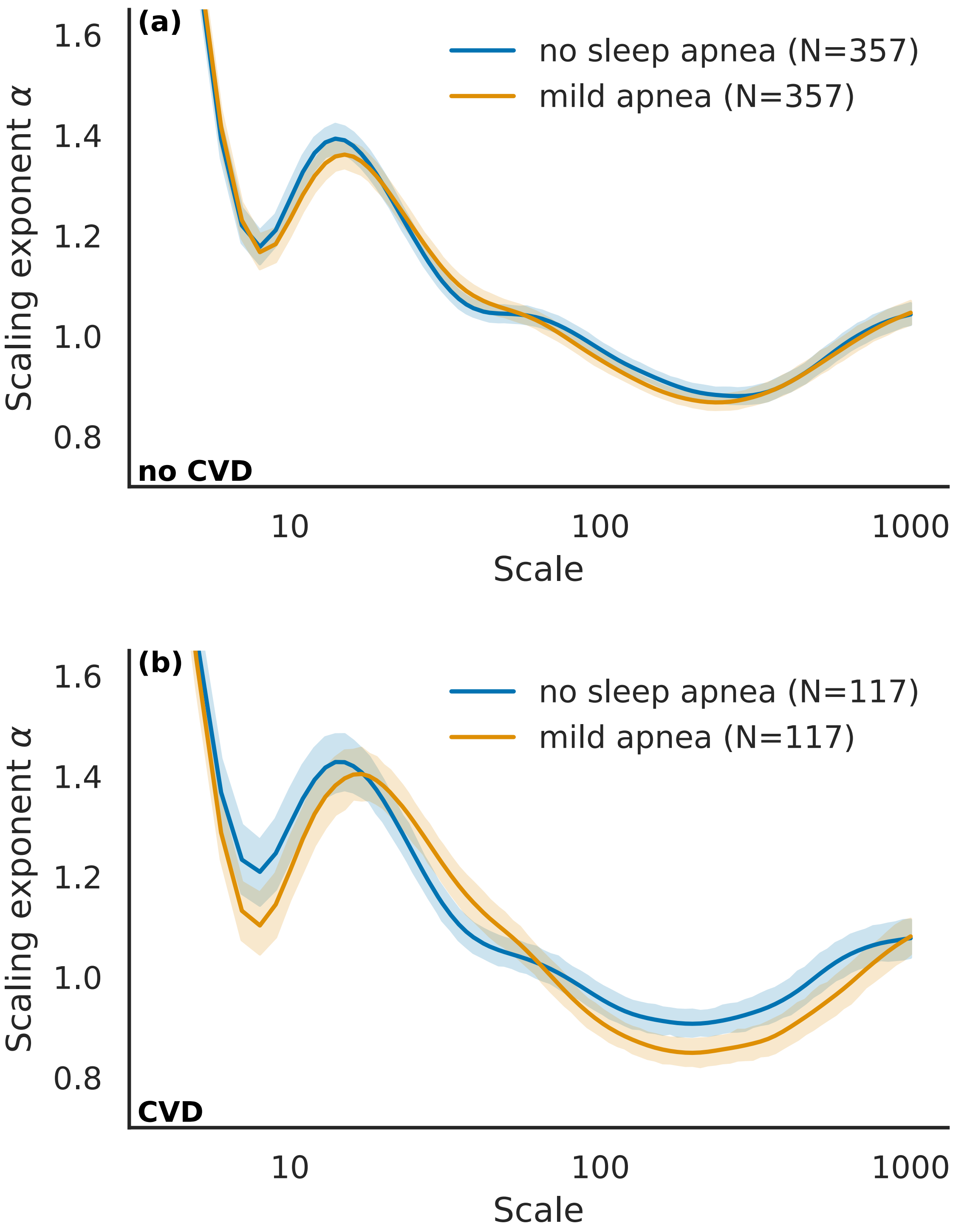

Figure S8: Scale-dependent DFA exponent (α) as a function of scale in male subjects with mild apnea and healthy controls, shown separately for those without (a) and with (b) cardiovascular disease (CVD). Participants with diabetes were excluded.

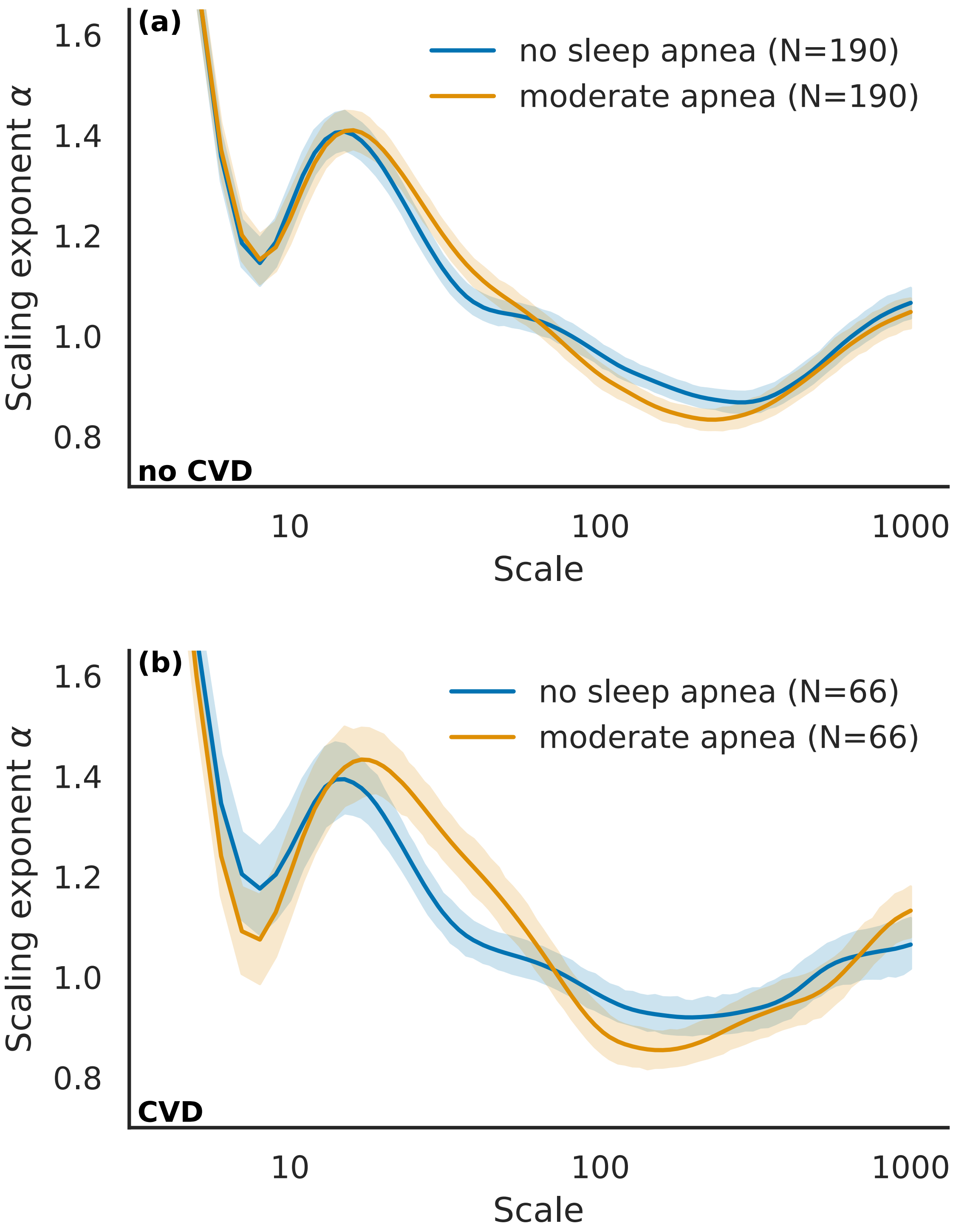

Figure S9: Scale-dependent DFA exponent (α) as a function of scale in male subjects with moderate apnea and healthy controls, shown separately for those without (a) and with (b) cardiovascular disease (CVD). Participants with diabetes were excluded.

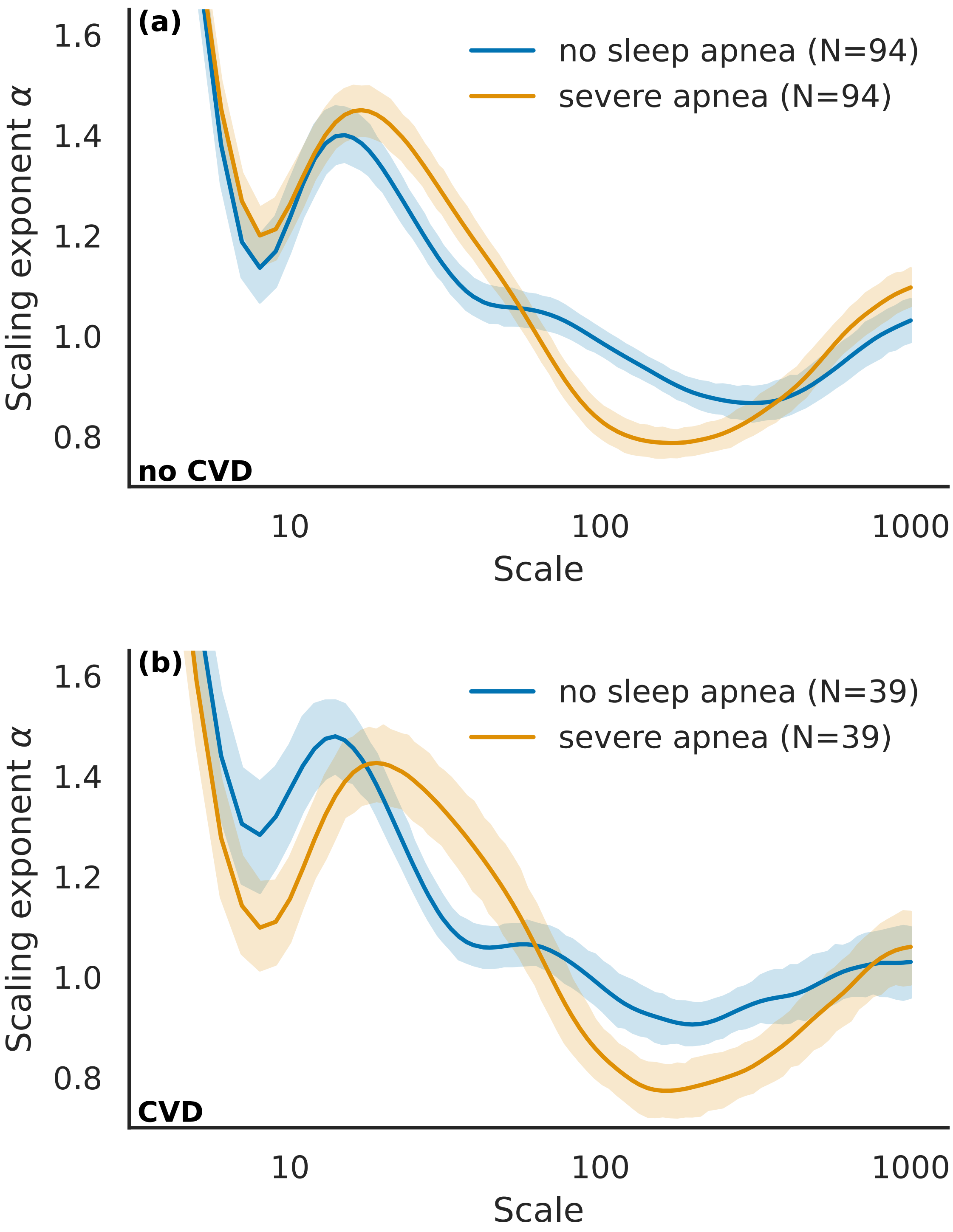

Figure S10: Scale-dependent DFA exponent (α) as a function of scale in male subjects with severe apnea and healthy controls, shown separately for those without (a) and with (b) cardiovascular disease (CVD). Participants with diabetes were excluded.

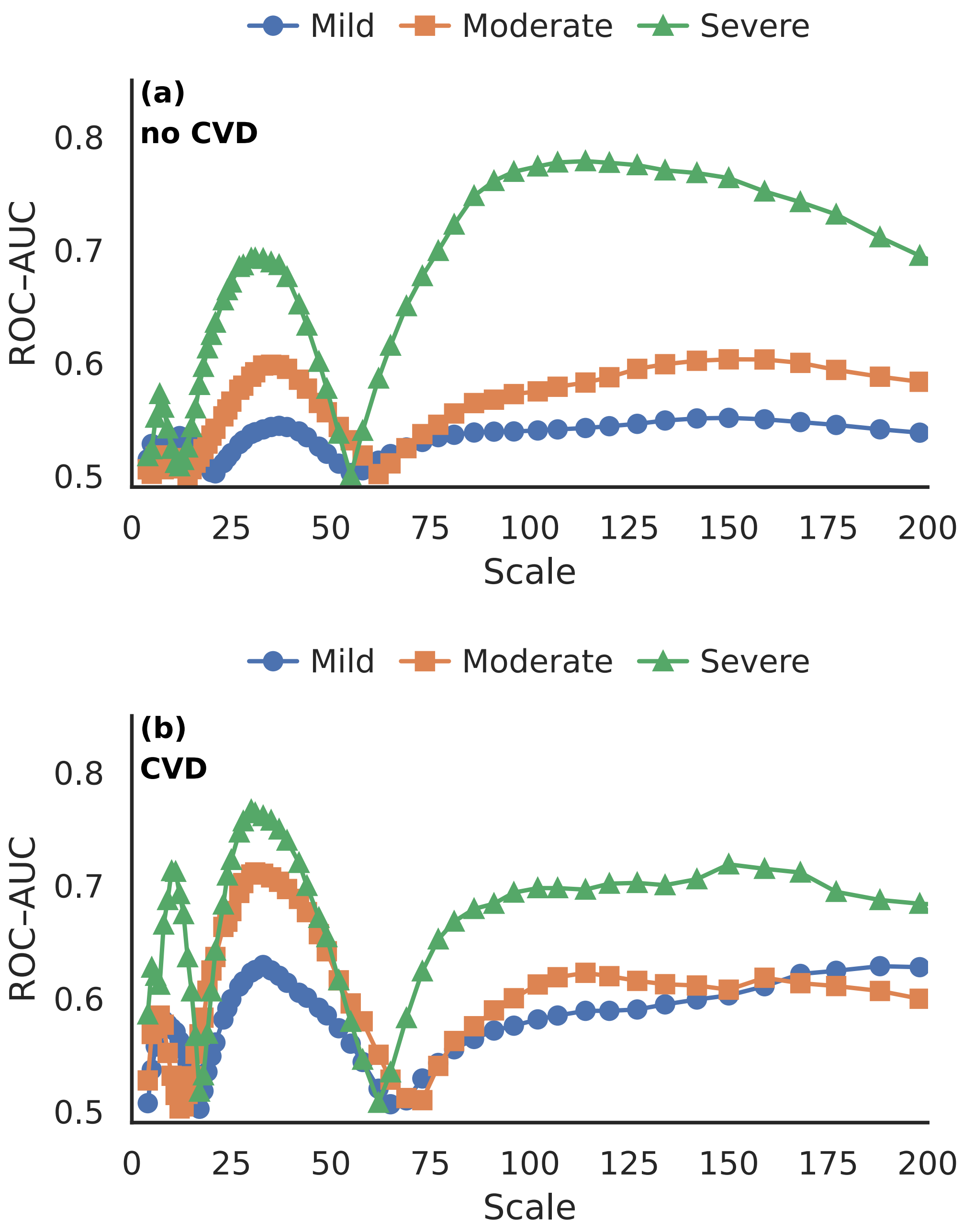

Figure S11: AUC of the scaling exponent (α) as a function of scale in male participants without (a) and with (b) cardiovascular disease (CVD), across all apnea severity levels. Participants with diabetes were excluded.

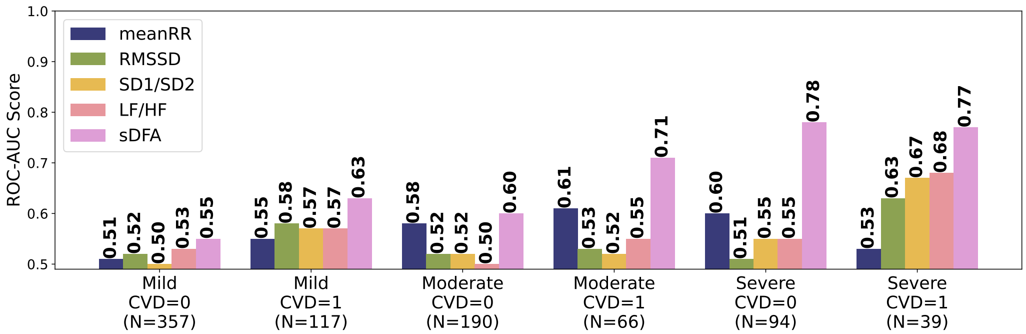

Figure S12: AUC scores of different HRV methods across apnea severity levels and CVD status in male subjects, with participants with diabetes excluded. Consistent with the full cohort, performance varies between subgroups, while sDFA outperforms conventional HRV metrics across all groups.

S4 Results for female participants without diabetes

Figures S13, S14, and S15 show the scaling exponent (α) as a function of scale in female subjects with mild, moderate, and severe apnea, respectively. The corresponding ROC–AUC values are presented in Fig. S16 and compared with conventional HRV metrics in Fig. S17. Subjects with diabetes were excluded from the analysis.

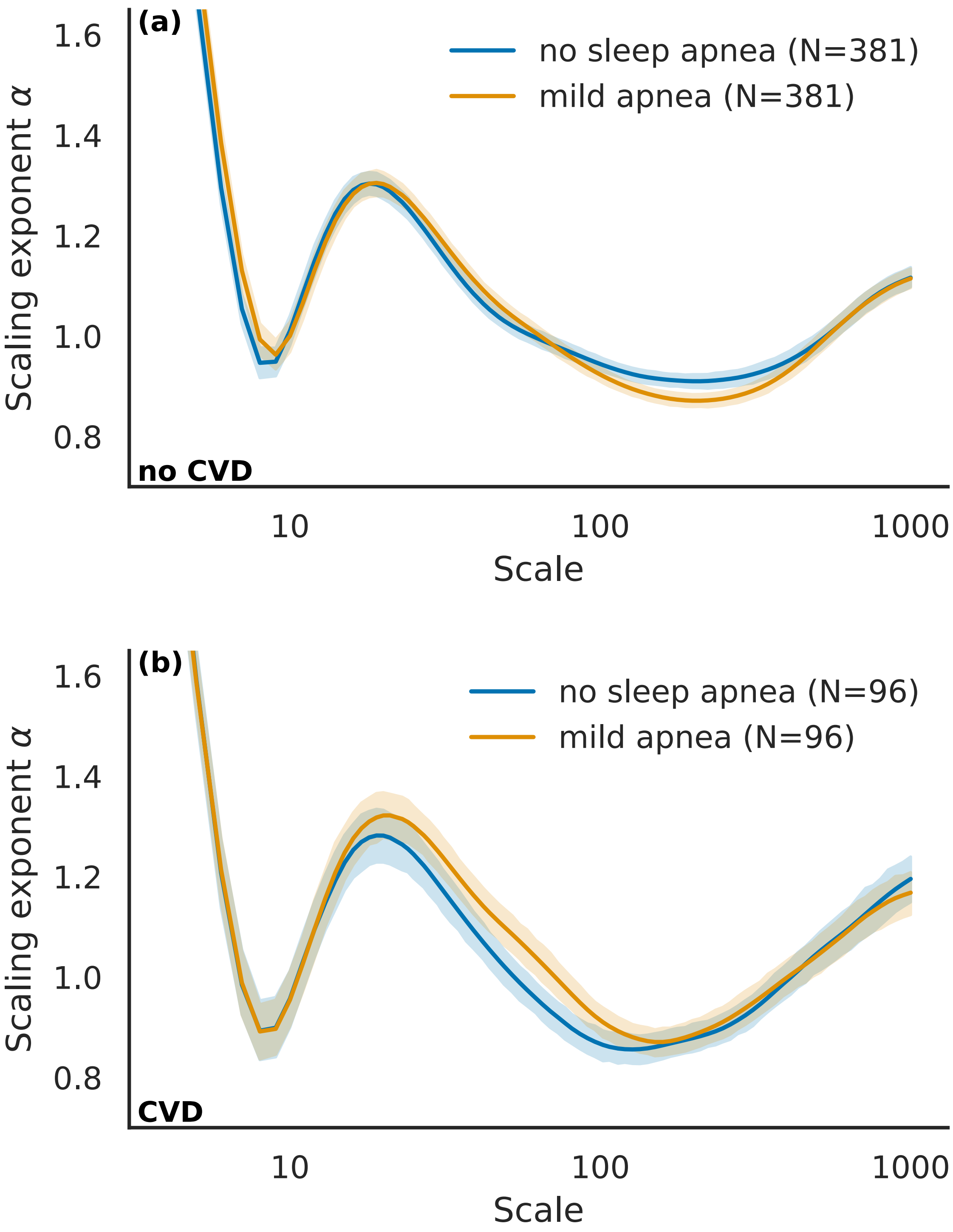

Figure S13: Scale-dependent DFA exponent (α) as a function of scale in female subjects with mild apnea and healthy controls, shown separately for those without (a) and with (b) cardiovascular disease (CVD). Participants with diabetes were excluded.

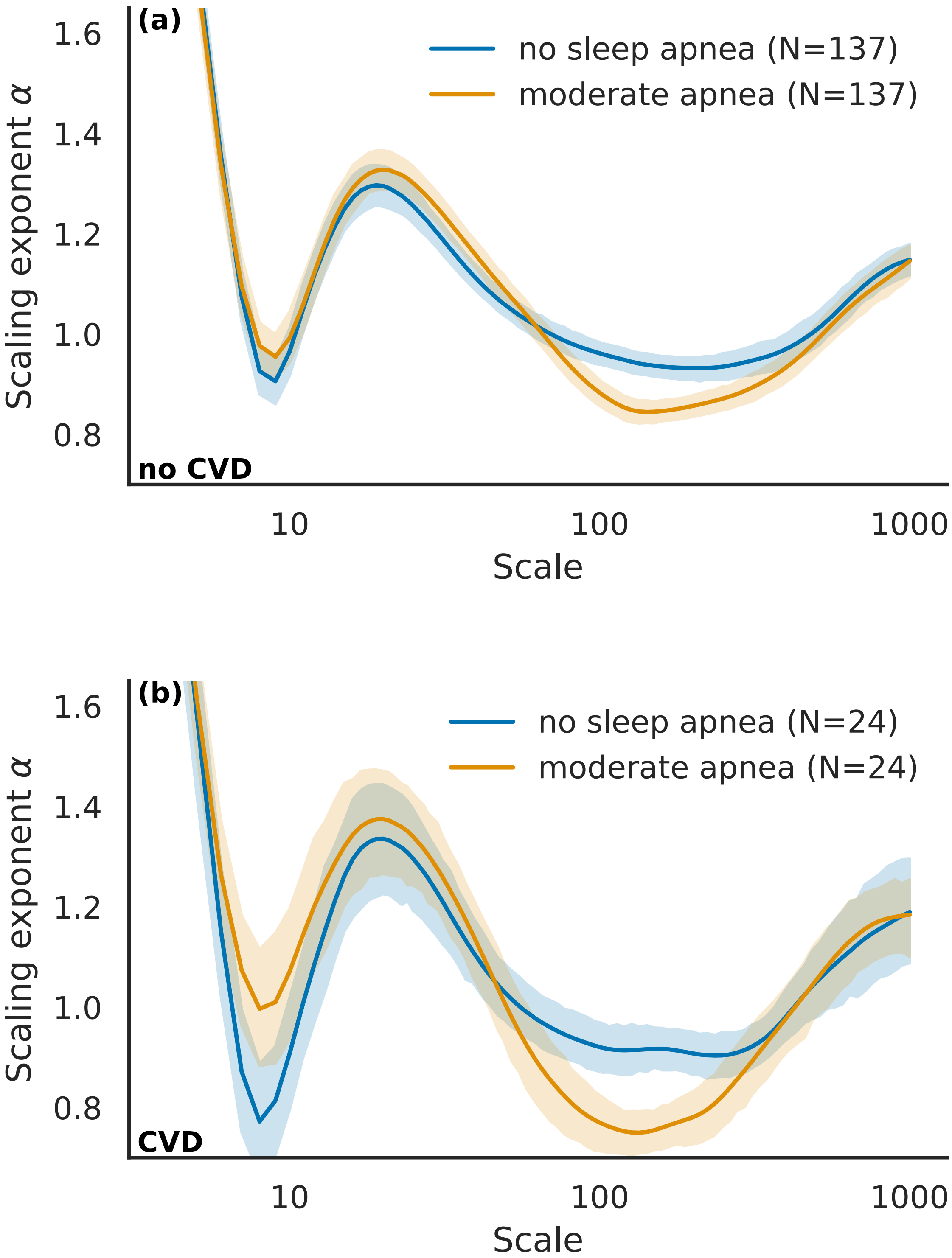

Figure S14: Scale-dependent DFA exponent (α) as a function of scale in female subjects with moderate apnea and healthy controls, shown separately for those without (a) and with (b) cardiovascular disease (CVD). Participants with diabetes were excluded.

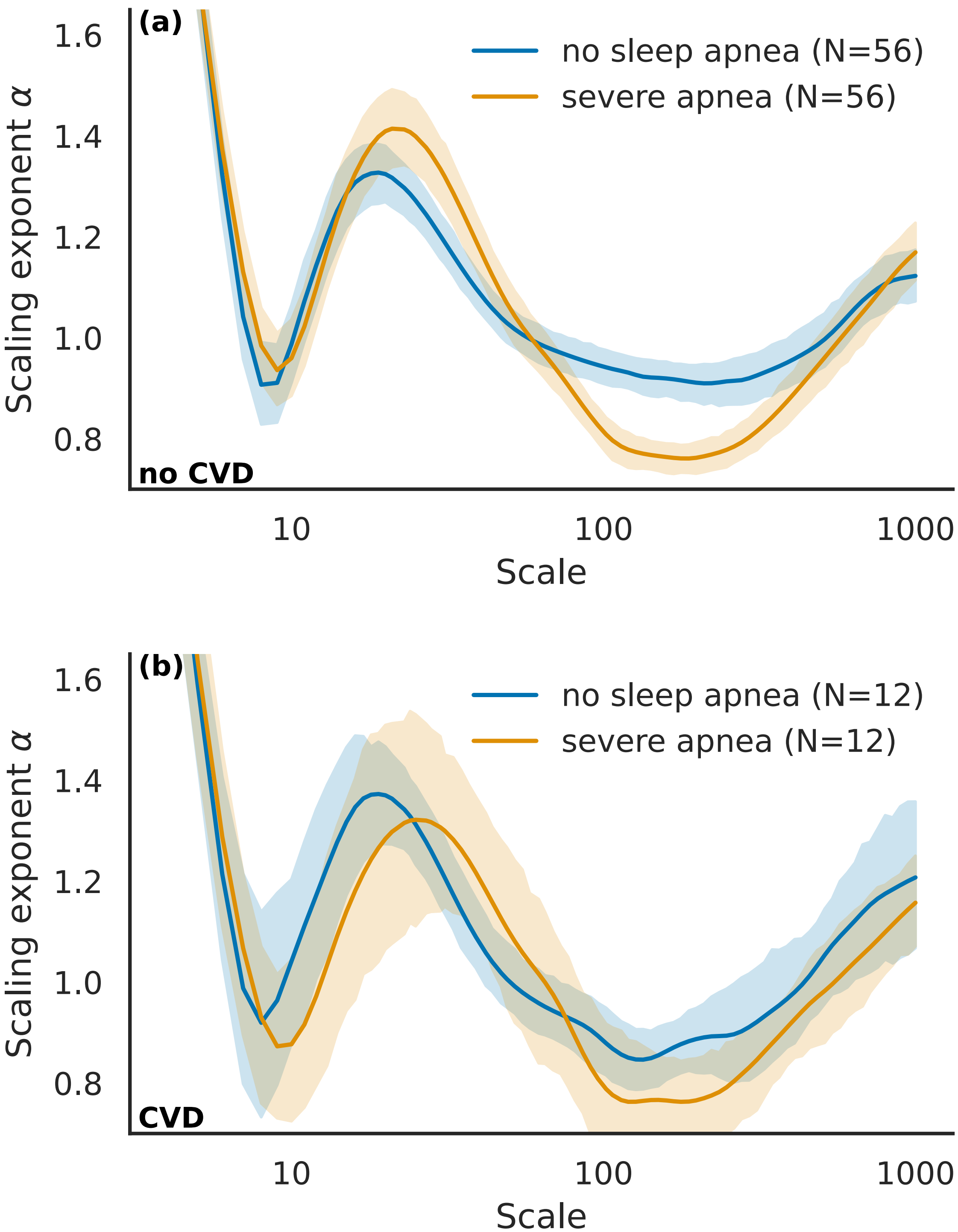

Figure S15: Scale-dependent DFA exponent (α) as a function of scale in female subjects with severe apnea and healthy controls, shown separately for those without (a) and with (b) cardiovascular disease (CVD). Participants with diabetes were excluded.

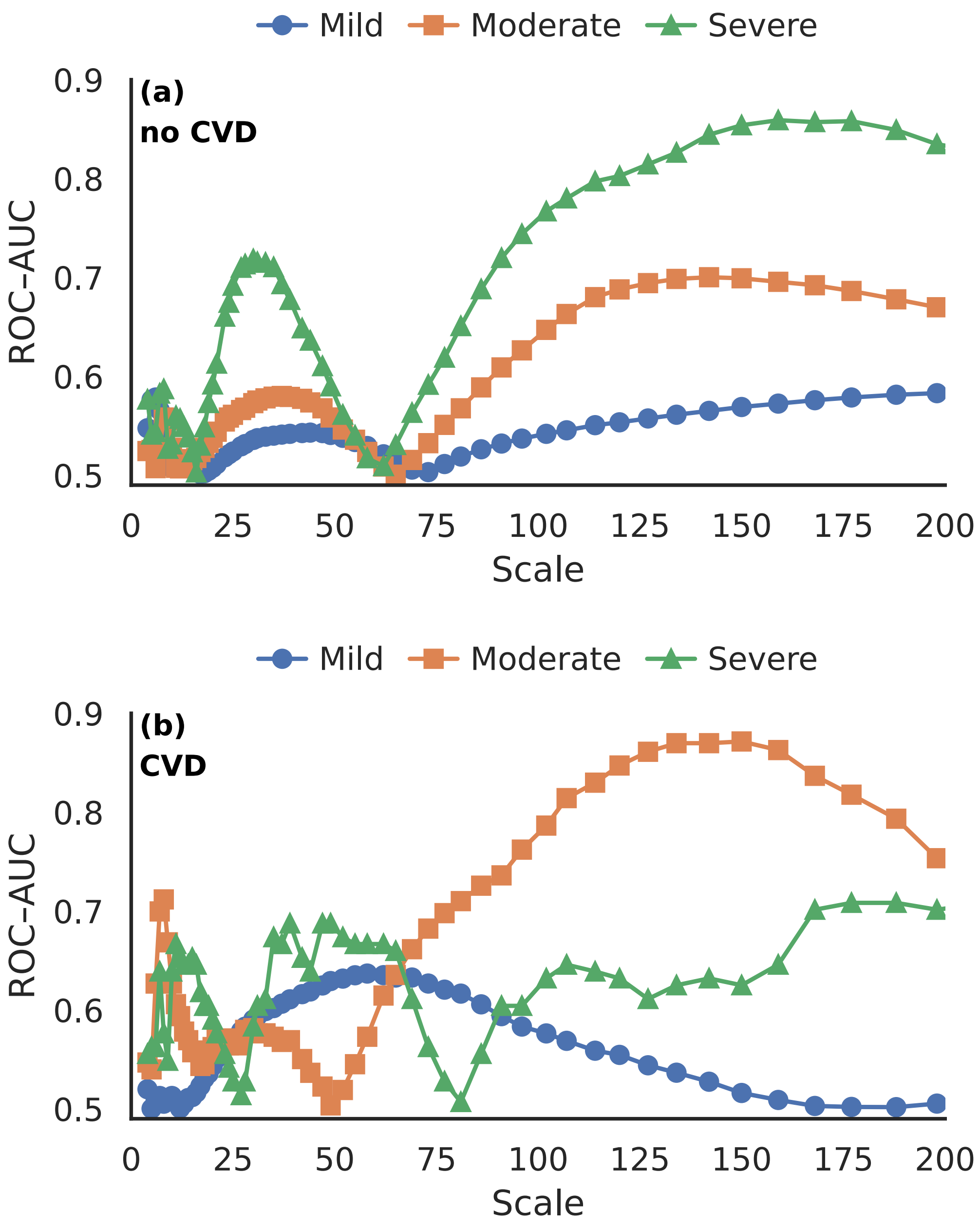

Figure S16: AUC of the scaling exponent (α) as a function of scale in female participants without (a) and with (b) cardiovascular disease (CVD), across all apnea severity levels. Participants with diabetes were excluded. Notably, in the CVD subgroup, moderate apnea shows higher AUC values than severe apnea.

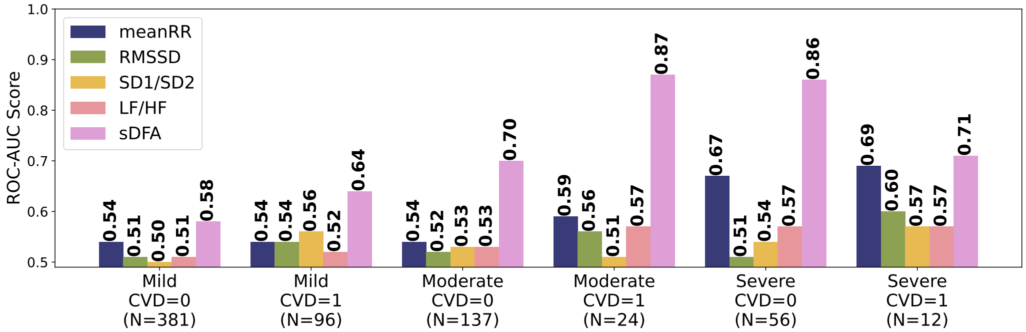

Figure S17: AUC scores of different HRV methods across apnea severity levels and CVD status in female subjects, with participants with diabetes excluded. Consistent with the results in males, performance varies between subgroups, while sDFA outperforms conventional HRV metrics across all groups. Notably, the moderate CVD = 1 subgroup shows comparatively strong performance; however, given the small sample size, this result should be interpreted with caution.

S5 Results for male participants results without medication

Figures S18, S19, and S20 show the scaling exponent (α) as a function of scale in male subjects with mild, moderate, and severe apnea, respectively. The corresponding ROC–AUC values are presented in Fig. S21 and compared with conventional HRV metrics in Fig. S22. Subjects receiving angiotensin-converting enzyme (ACE) inhibitors, beta-blockers, or calcium-channel blockers (CCIR, CCBSR, or CCBT) were excluded from the analysis.

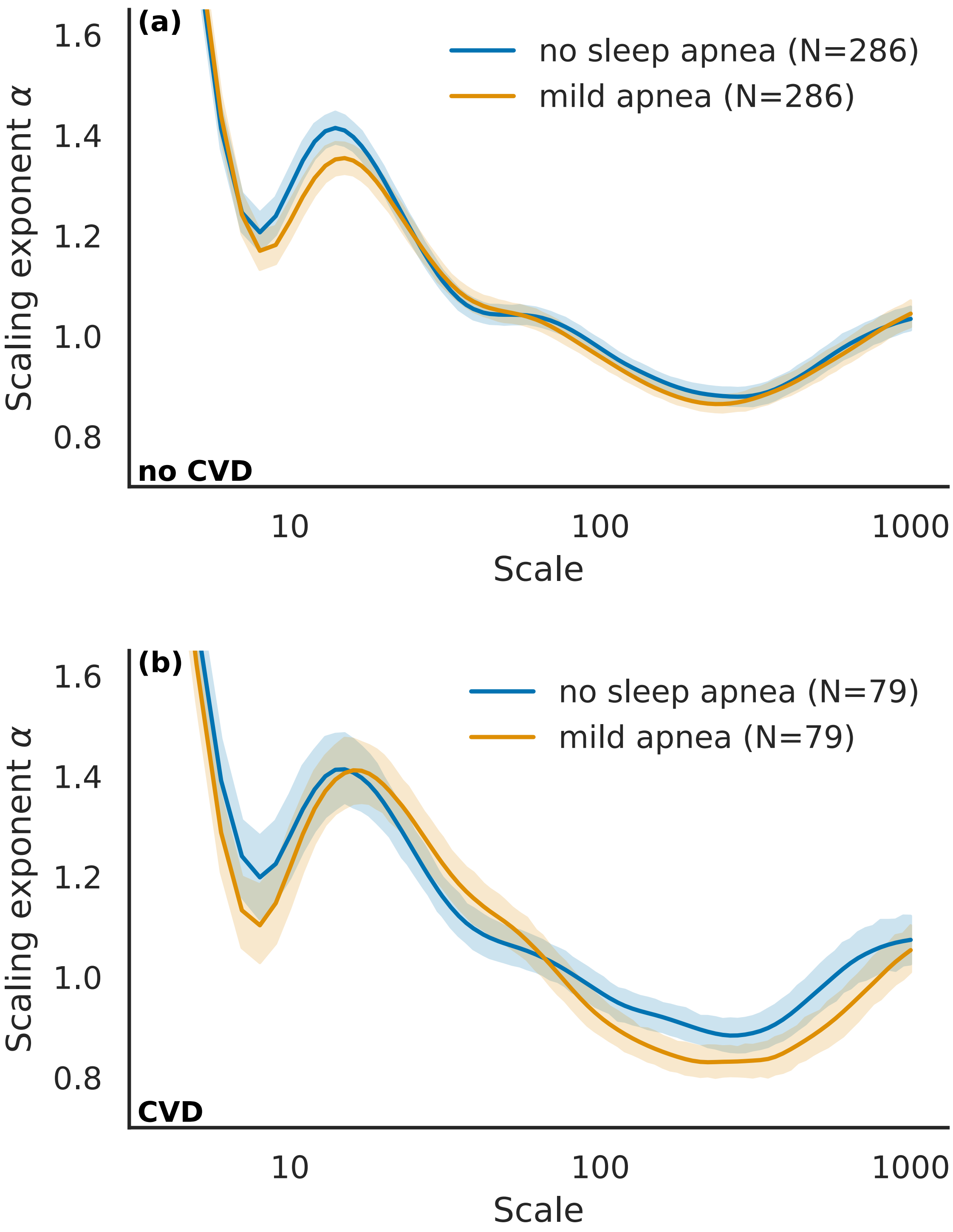

Figure S18: Scale-dependent DFA exponent (α) as a function of scale in male subjects with mild apnea and healthy controls, shown separately for those without (a) and with (b) cardiovascular disease (CVD). Participants using relevant medication were excluded.

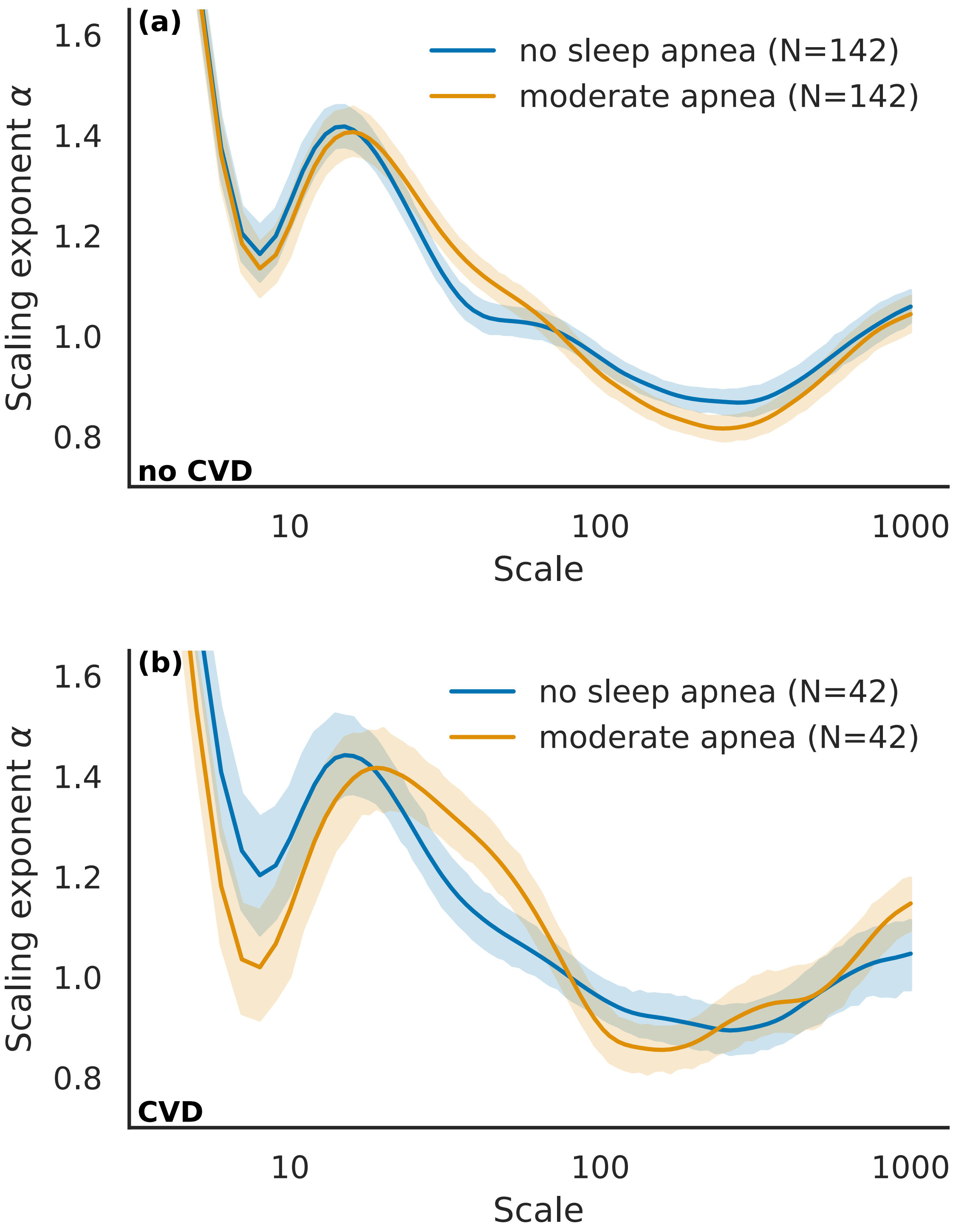

Figure S19: Scale-dependent DFA exponent (α) as a function of scale in male subjects with moderate apnea and healthy controls, shown separately for those without (a) and with (b) cardiovascular disease (CVD). Participants using relevant medication were excluded.

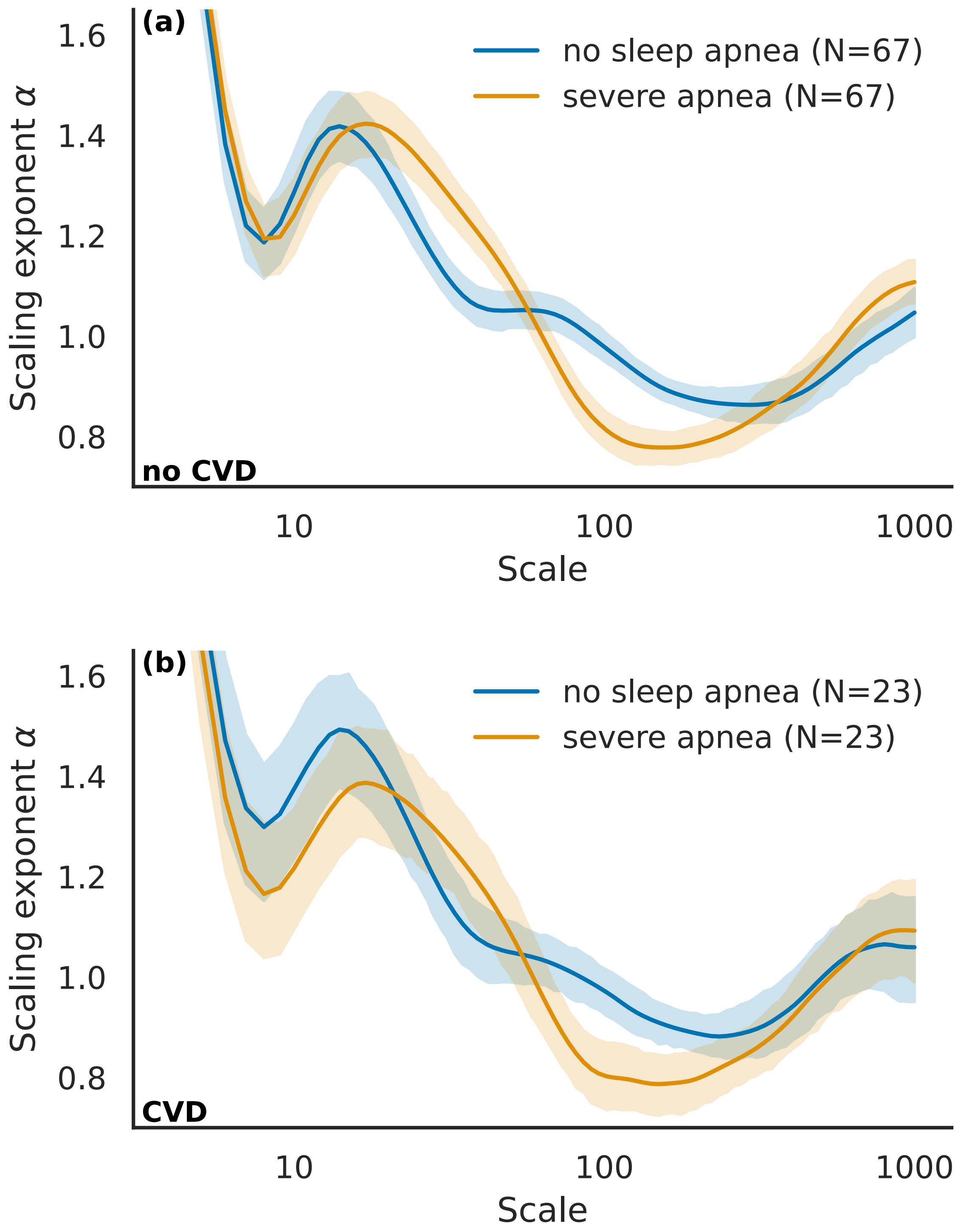

Figure S20: Scale-dependent DFA exponent (α) as a function of scale in male subjects with severe apnea and healthy controls, shown separately for those without (a) and with (b) cardiovascular disease (CVD). Participants using relevant medication were excluded.

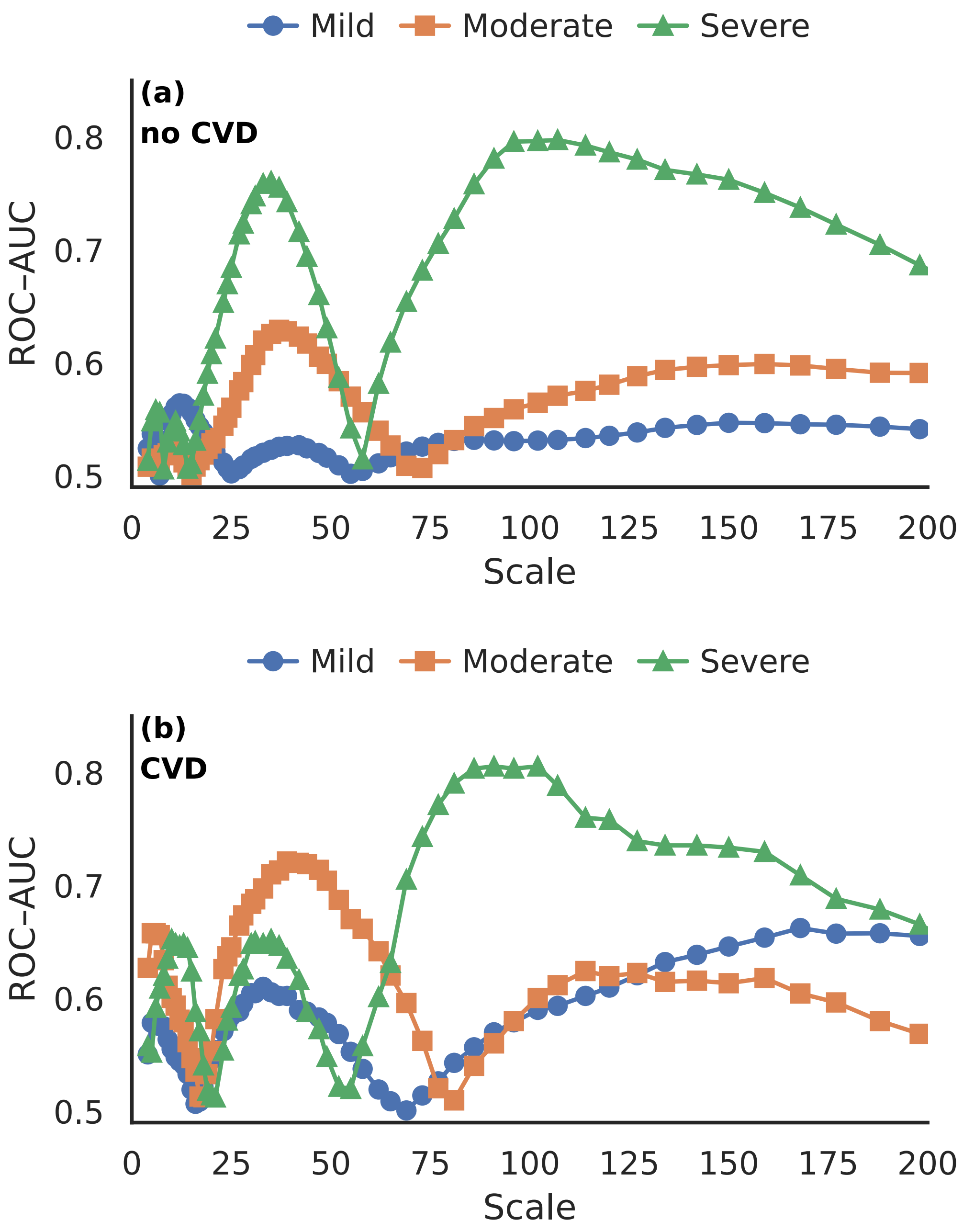

Figure S21: AUC of the scaling exponent (α) as a function of scale in male participants without (a) and with (b) cardiovascular disease (CVD), across all apnea severity levels. Participants using relevant medication were excluded.

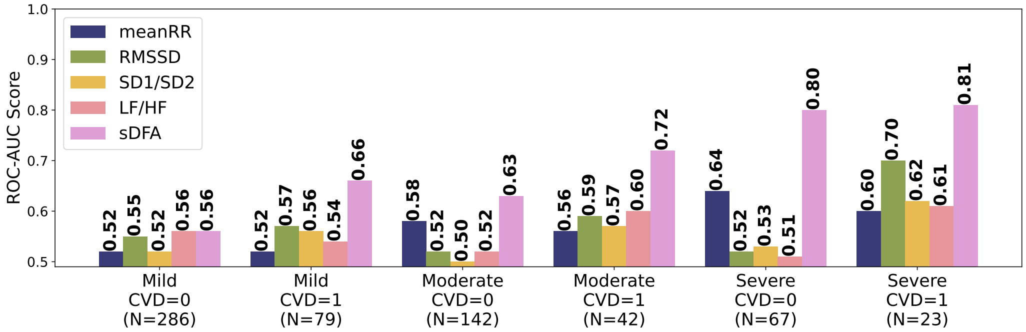

Figure S22: AUC scores of different HRV methods across apnea severity levels and CVD status in male subjects after exclusion of participants using relevant medication. Consistent with previous results, performance varies between subgroups, while sDFA outperforms conventional HRV metrics across all groups. The reduced sample size should be considered when interpreting these findings.

S6 Results for female participants results without medication

Figures S23, S24, and S25 show the scaling exponent (α) as a function of scale in female subjects with mild, moderate, and severe apnea, respectively. The corresponding ROC–AUC values are presented in Fig. S26 and compared with conventional HRV metrics in Fig. S27. Subjects receiving angiotensin-converting enzyme (ACE) inhibitors, beta-blockers, or calcium-channel blockers (CCIR, CCBSR, or CCBT) were excluded from the analysis.

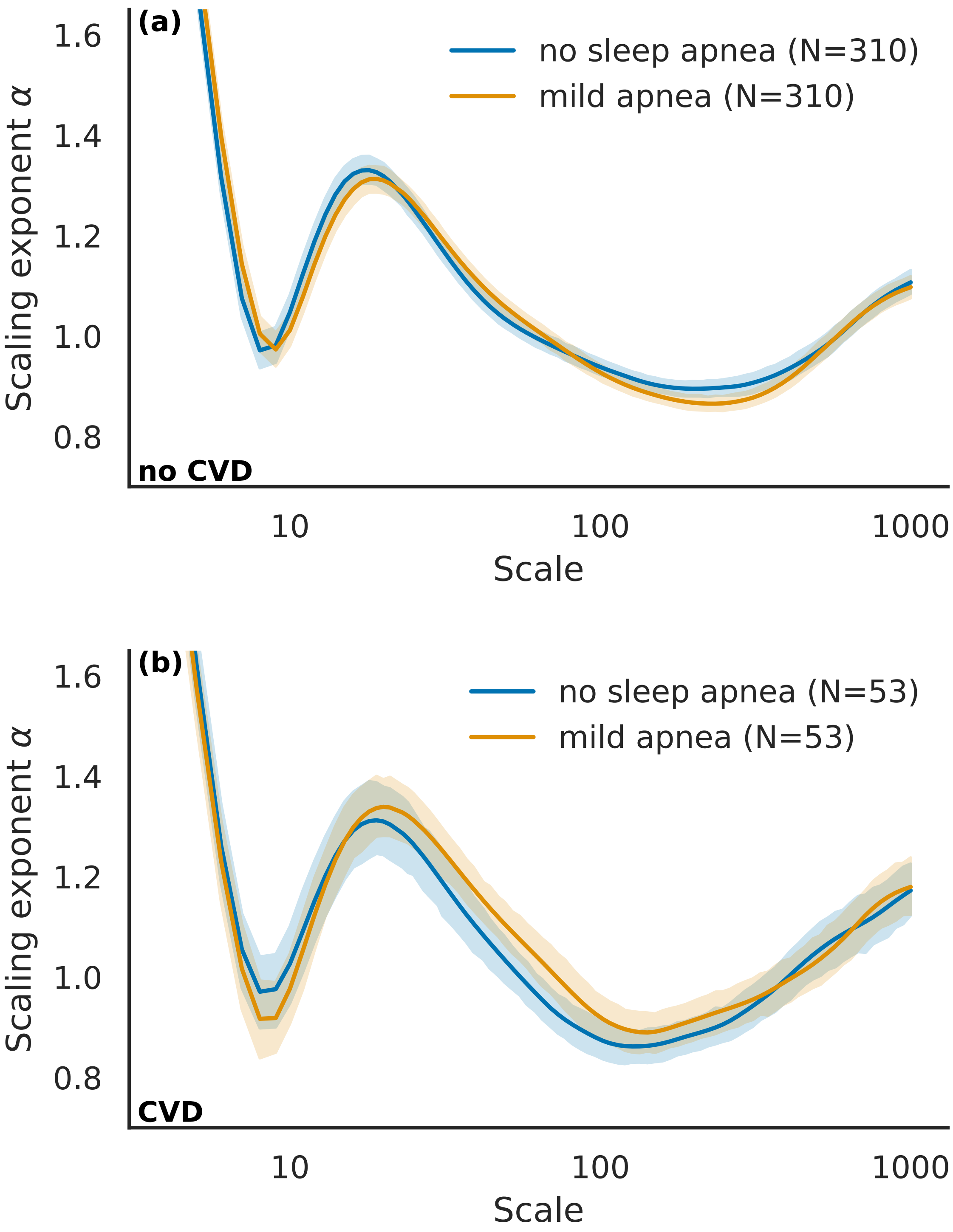

Figure S23: Scale-dependent DFA exponent (α) as a function of scale in female subjects with mild apnea and healthy controls, shown separately for those without (a) and with (b) cardiovascular disease (CVD). Participants using relevant medication were excluded.

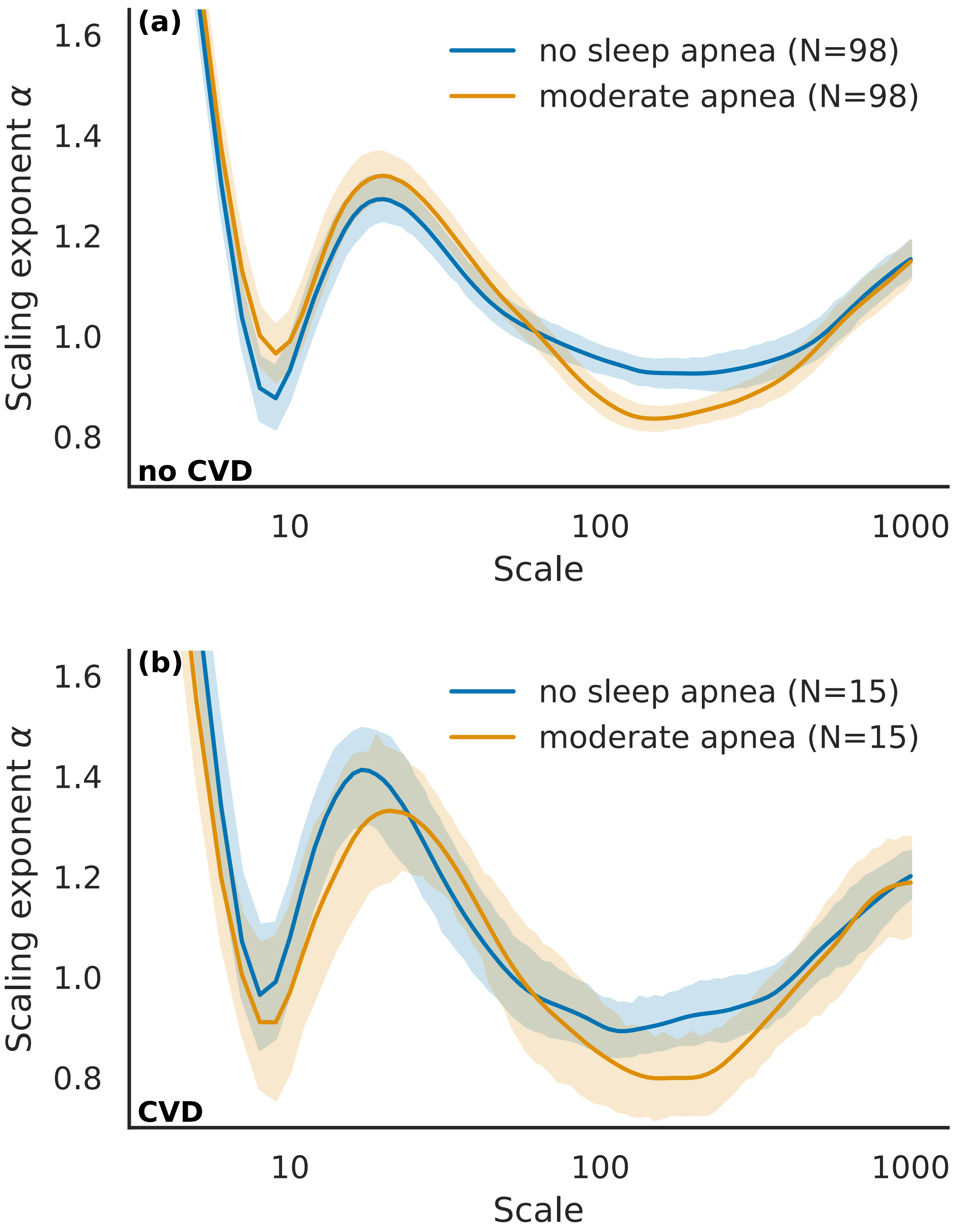

Figure S24: Scale-dependent DFA exponent (α) as a function of scale in female subjects with moderate apnea and healthy controls, shown separately for those without (a) and with (b) cardiovascular disease (CVD). Participants using relevant medication were excluded.

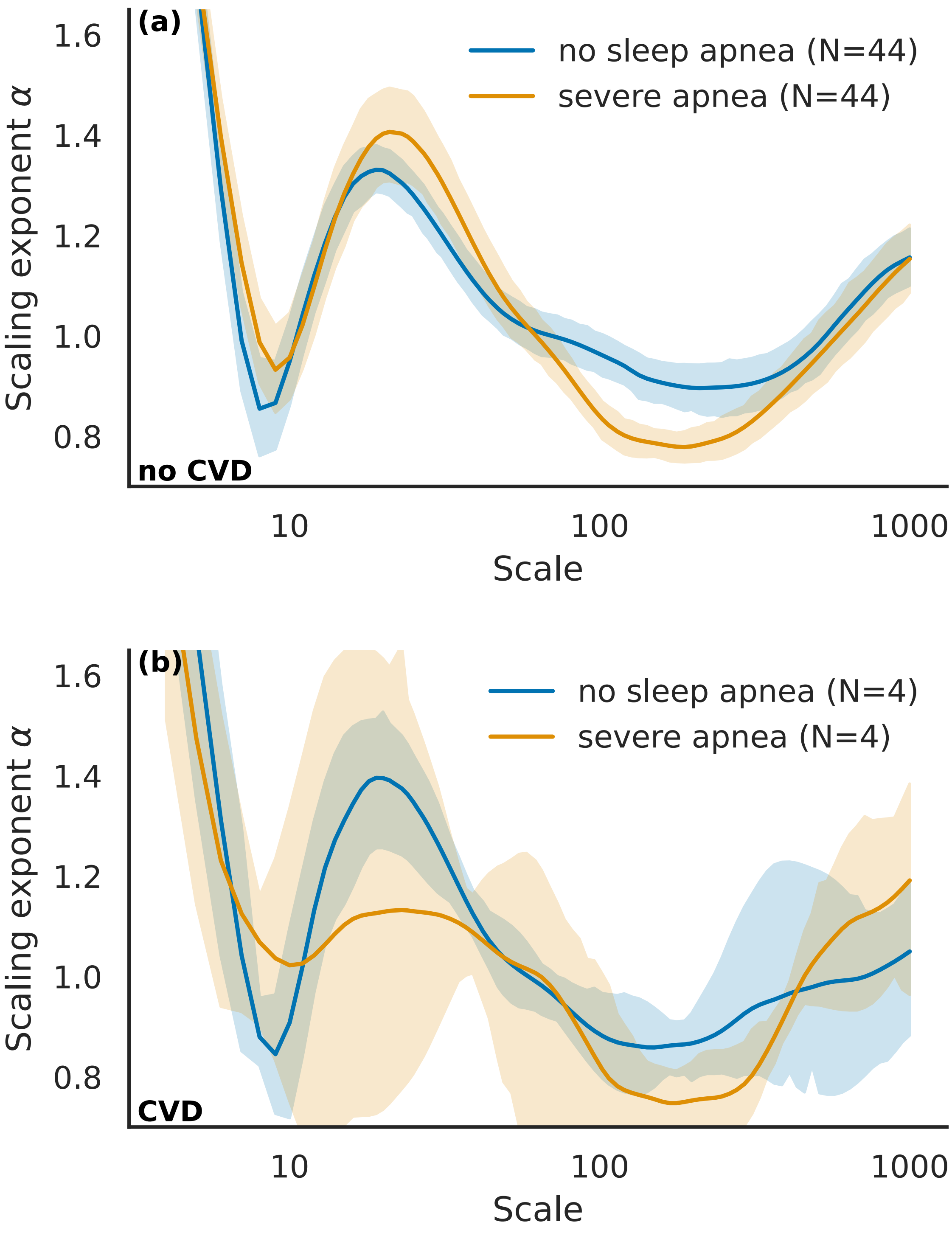

Figure S25: Scale-dependent DFA exponent (α) as a function of scale in female subjects with severe apnea and healthy controls, shown separately for those without (a) and with (b) cardiovascular disease (CVD). Participants using relevant medication were excluded.

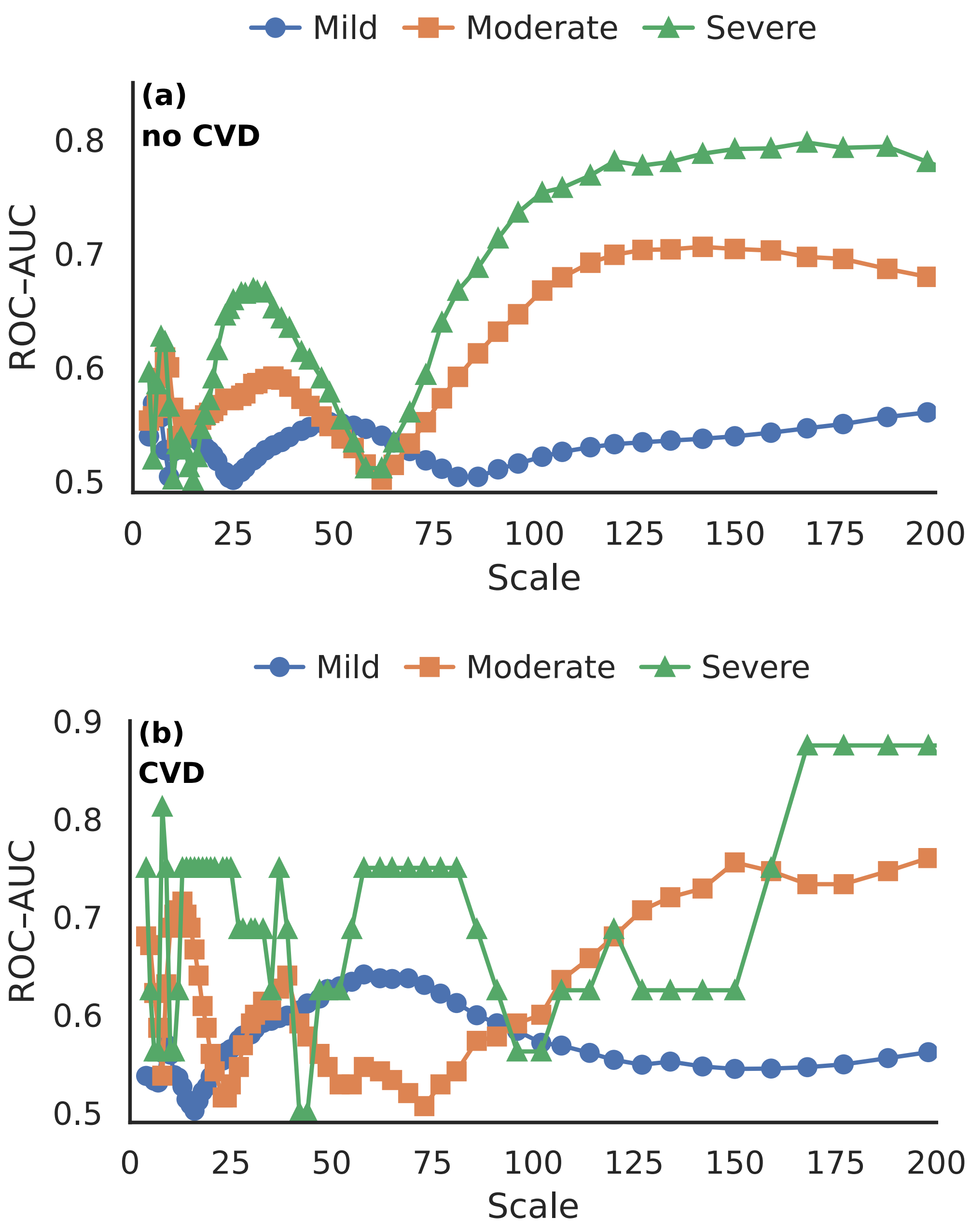

Figure S26: AUC of the scaling exponent (α) as a function of scale in male participants without (a) and with (b) cardiovascular disease (CVD), across all apnea severity levels.

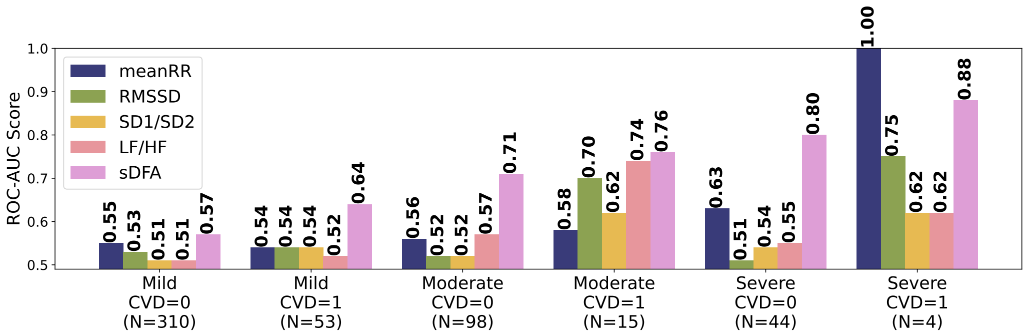

Figure S27: AUC scores of different HRV methods across apnea severity levels and CVD status in male subjects, with medicated individuals excluded. Consistent with the other results, performance varies across subgroups, while sDFA generally outperforms conventional HRV metrics. The only exception is the severe CVD = 1 subgroup; however, as this group includes only four subjects, these results should be interpreted with caution.

S7 ROC-AUC curves

The following figures show the best-performing ROC curves for males with severe apnea without cardiovascular disease (Fig. S28) and with cardiovascular disease (Fig. S29). The selected operating point is highlighted with a red dot, and the corresponding sensitivity and specificity values are reported.

Males with severe apnea and no cardiovascular disease showed a sensitivity of 60% and a specificity of 85% (Fig. S28). In contrast, males with severe apnea and cardiovascular disease showed a sensitivity of 64% and a specificity of 81% (Fig. S29).

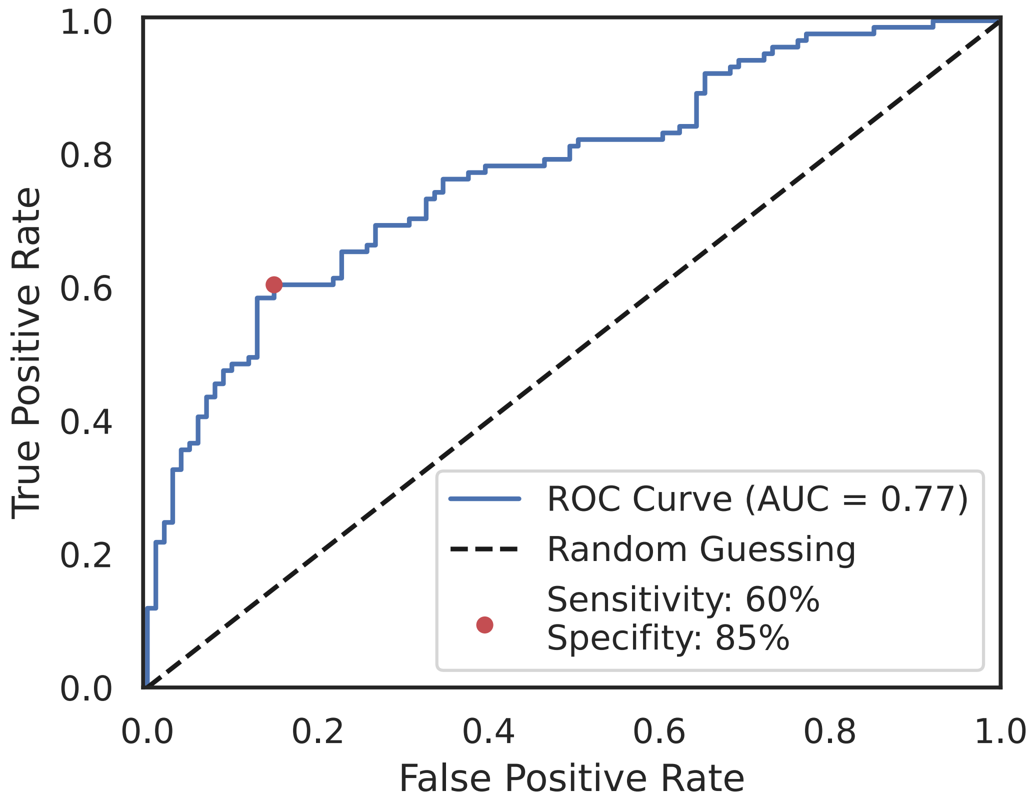

Figure S28: Receiver operating characteristic curve for males with severe apnea and no cardiovascular disease.

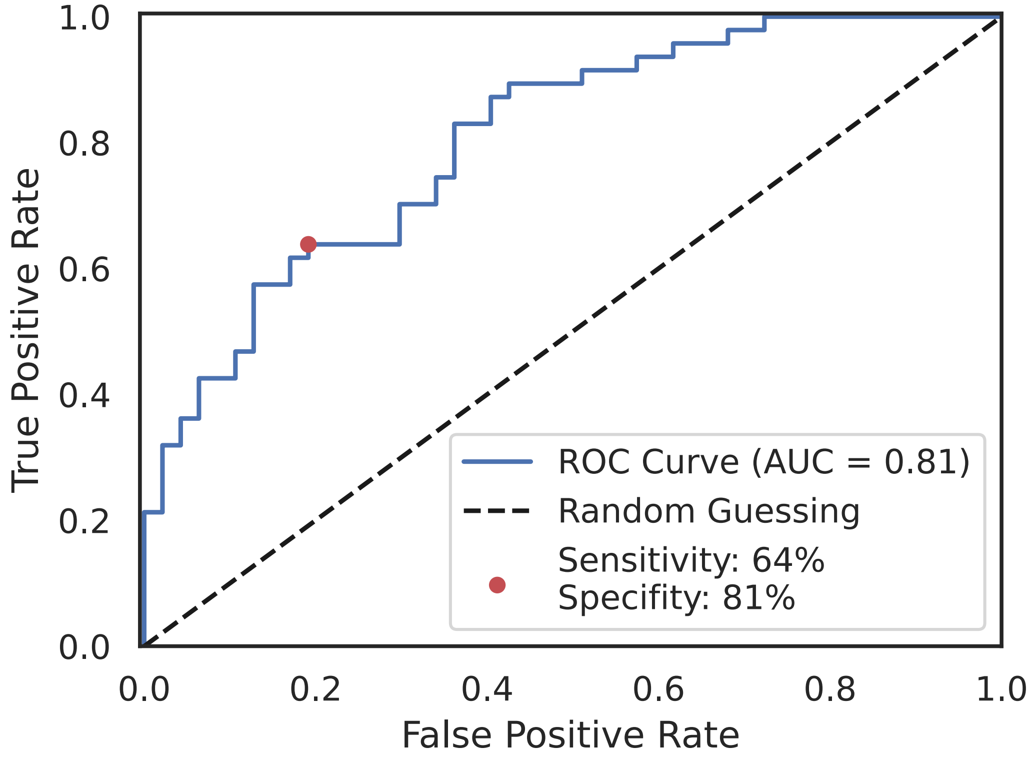

Figure S29: Receiver operating characteristic curve for males with severe apnea and cardiovascular disease.
